# RNA Exosome Complex-Mediated Control of Redox Status in Pluripotent Stem Cells

**DOI:** 10.1016/j.stemcr.2017.08.024

**Published:** 2017-10-10

**Authors:** Maria Skamagki, Cheng Zhang, Christian A. Ross, Aparna Ananthanarayanan, Zhong Liu, Quanhua Mu, Uttiya Basu, Jiguang Wang, Rui Zhao, Hu Li, Kitai Kim

**Affiliations:** 1 Cancer Biology and Genetics Program, The Center for Cell Engineering, The Center for Stem Cell Biology, Memorial Sloan-Kettering Cancer Center, Sloan-Kettering Institute for Cancer Research, New York, NY 10065, USA; 2 Department of Cell and Developmental Biology, Weill Medical College of Cornell University, New York, NY 10065, USA; 3 Department of Molecular Pharmacology & Experimental Therapeutics, Center for Individualized Medicine, Mayo Clinic College of Medicine, Rochester, MN 55902, USA; 4 Department of Biochemistry and Molecular Genetics, Stem Cell Institute, University of Alabama at Birmingham, Birmingham, AL 35294, USA; 5 Divisions of Life Science, Department of Chemical and Biomedical Engineering, School of Engineering, The Hong Kong University of Science and Technology, Clear Water Bay, Kowloon, Hong Kong; 6 Department of Microbiology and Immunology, College of Physicians and Surgeons, Columbia University, New York, NY 10032, USA

**Keywords:** RNA exosome complex, GPX2, glutathione, reactive oxygen species, ROS, homeostatic balance, DNA damage response, pluripotent stem cells, induced pluripotent stem cells, aging

## Abstract

The RNA exosome complex targets AU-rich element (ARE)-containing mRNAs in eukaryotic cells. We identified a transcription factor, ZSCAN10, which binds to the promoters of multiple RNA exosome complex subunits in pluripotent stem cells to maintain subunit gene expression. We discovered that induced pluripotent stem cell clones generated from aged tissue donors (A-iPSC) show poor expression of ZSCAN10, leading to poor RNA exosome complex expression, and a subsequent elevation in ARE-containing RNAs, including glutathione peroxidase 2 (*Gpx2*). Excess GPX2 leads to excess glutathione-mediated reactive oxygen species scavenging activity that blunts the DNA damage response and apoptosis. Expression of ZSCAN10 in A-iPSC recovers RNA exosome gene expression, the DNA damage response, and apoptosis. These findings reveal the central role of ZSCAN10 and the RNA exosome complex in maintaining pluripotent stem cell redox status to support a normal DNA damage response.

## Highlights

• ZSCAN10 binds to transcription start sites of multiple RNA exosome complex subunits
• iPSCs with poor ZSCAN10 expression cause poor expression of RNA exosome complex subunits
• Consequently, poor expression of RNA exosome complex core subunits elevates ARE-containing RNAs, including GPX2
• Higher GPX2 blunts DNA damage response by homeostatic imbalance of ROS and glutathione

## Graphical Abstract

**Figure:**
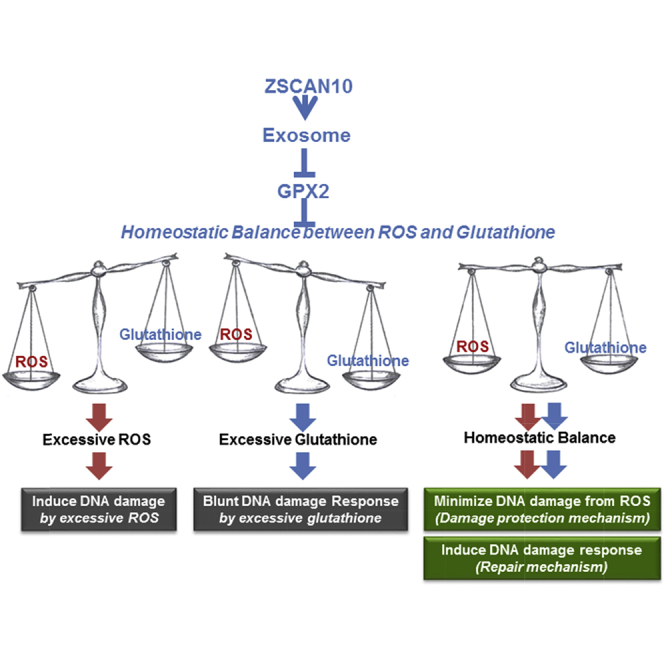

## Teaser

Skamagki et al. demonstrate that ZSCAN10 regulates GPX2 expression by controlling RNA exosome complex in pluripotent stem cells. Low ZSCAN10 results in higher GPX2, which blunts DNA damage response by unbalancing ROS and glutathione, which may affect clinical utility.

## Introduction

The RNA exosome complex is a central ring structure of six core proteins and three RNA-binding domain-containing core proteins (Kilchert et al., 2016, Makino et al., 2015) that removes aberrantly accumulated RNA transcripts to prevent events such as altered splicing (Coy et al., 2013), autoimmune response activation (Eckard et al., 2014), and genomic instability caused by RNA-DNA hybridization (Wahba et al., 2013). The RNA exosome complex is highly conserved in eukaryotic cells and functions in both the nucleus and the cytoplasm (Schmid and Jensen, 2008). Recent reports have shown that the RNA exosome complex targets specific RNA transcripts in response to environmental changes during embryo development (Kilchert et al., 2015). In the cytoplasm, the exosome complex interacts with RNA at A + U sequence-rich elements (AREs), causing rapid degradation of target RNA with significant specificity (Chen et al., 2001, Doma and Parker, 2006). Recently, the targeting specificity of the RNA exosome complex was studied in embryonic stem cells (ESCs) by transcriptome analysis (Pefanis et al., 2015). That study showed that the exosome regulates long non-coding RNA transcripts that function as enhancer RNAs to control gene transcription. We found that induced pluripotent stem cells (iPSCs) (Takahashi and Yamanaka, 2006) derived from older donor cells (A-iPSCs; using mouse skin fibroblasts from donors 1.4 years of age) retain an aging-associated epigenetic signature that is not present in iPSCs derived from young donor cells (Y-iPSCs; using mouse skin fibroblasts from E15.5 embryos to 5-day-old neonates) (Skamagki et al., 2017, Kim et al., 2010, Kim et al., 2011). A-iPSCs show transcriptional alterations compared with Y-iPSCs, including poor expression of the pluripotent factor ZSCAN10. ZSCAN10 is a known zinc finger transcription factor specifically expressed in ESCs and an integrated part of the transcriptional regulatory network with SOX2, OCT4, and NANOG (Yu et al., 2009, Zhang et al., 2006). In this report, we found that ZSCAN10 directly binds to the promoters of RNA exosome subunits to stimulate their expression, and that A-iPSCs show poor expression of core RNA exosome subunits due to poor ZSCAN10 expression, which could result in dysfunctional RNA exosome function in A-iPSCs (Figure S1). We utilized A-iPSCs to test the hypothesis that ZSCAN10 regulates gene transcription in PSCs via the RNA exosome complex, specifically focusing on glutathione peroxidase 2 (GPX2) as a downstream target that influences the DNA damage response pathway.

## Results

### GPX2 Modulates Glutathione Activity and Redox Status in Induced Pluripotent Stem Cells from Young and Aged Donors

**Figure 1:**
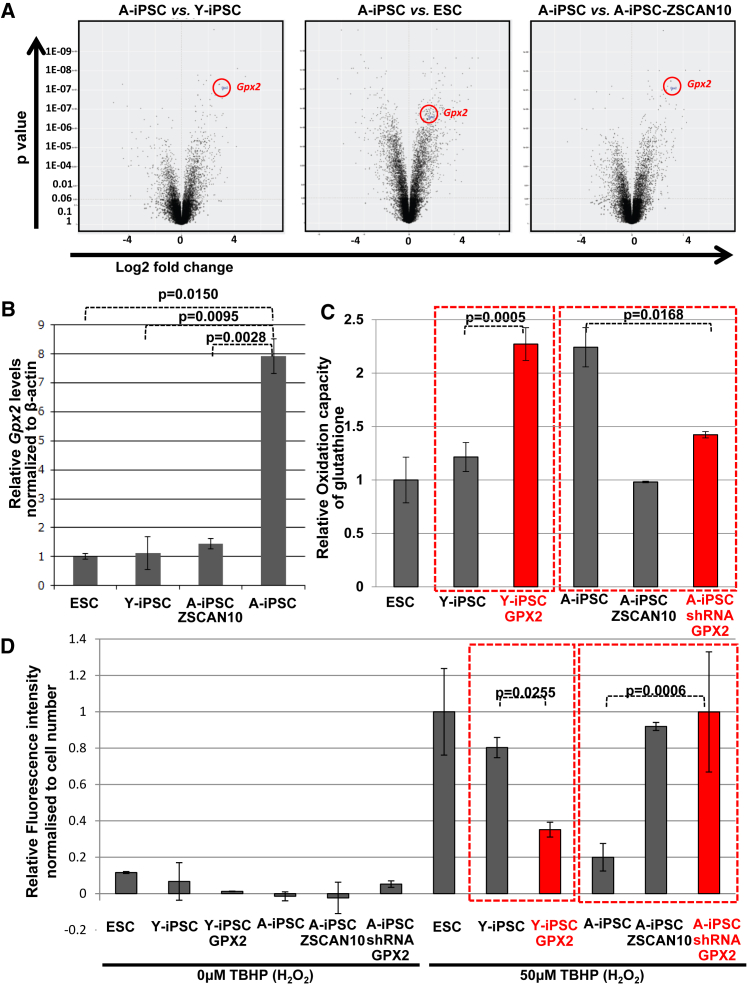
Imbalance of H_2_O_2_/Glutathione Homeostasis in A-iPSCs and Recovery by ZSCAN10 via Reduction of Excessively Activated GPX2 (A) Altered *Gpx2* expression from a comparative gene expression analysis (microarray). *Gpx2* is differentially expressed among the different cell lines (also refer to Figure S4A). At least two clonal repeats (Y-iPSC = 2, A-iPSC = 2, A-iPSC-ZSCAN10 = 4, ESC = 2) were included in the analysis. (B) qPCR for *Gpx2* indicating increased expression of GPX2 in A-iPSCs and downregulation by ZSCAN10 expression. Error bars indicate SEM (n = 4). (C) Quantification of reduced glutathione in ESCs, Y-iPSCs, Y-iPSC-GPX2, A-iPSCs, A-iPSC-ZSCAN10, and A-iPSC-shRNA-GPX2. Increased oxidation capacity of glutathione in A-iPSCs is recovered by reduction of GPX2 via shRNA-GPX2. Conversely, GPX2 overexpression in Y-iPSCs induces elevated glutathione oxidation capacity. Mean ± SD is plotted for four replicates from each condition (n = 4). Statistical significance was determined by two-sided t test. (D) H_2_O_2_ scavenging activity in ESCs, Y-iPSCs, Y-iPSC-GPX2, A-iPSCs, A-iPSC-ZSCAN10, and A-iPSC-shRNA-GPX2. Increased H_2_O_2_ scavenging activity in A-iPSCs is recovered by shRNA-GPX2 expression. Elevated H_2_O_2_ scavenging activity seen in A-iPSCs can be recapitulated in Y-iPSC by GPX2 overexpression. Mean ± SD is plotted for four replicates from each condition (n = 4). Statistical significance was determined by two-sided t test.

We performed a comparative gene expression analysis of ESCs, Y-iPSCs, A-iPSCs, and A-iPSCs expressing ZSCAN10 to identify candidate genes that were up- or downregulated in A-iPSCs compared with A-iPSC-ZSCAN10, and that were expressed at similar levels in A-iPSC-ZSCAN10, ESCs, and Y-iPSCs. We found that the *Gpx2* gene is highly expressed only in A-iPSCs and expression was normalized by ZSCAN10 expression (Figure 1A and S4A), as confirmed by qPCR (Figure 1B). GPX2 supports reactive oxygen species (ROS) scavenging by increasing the reduced form of glutathione (active form) from oxidized glutathione (Chu et al., 2004). We tested the possibility that increased expression of GPX2 in A-iPSCs could induce an imbalance in glutathione-ROS homeostasis. Overexpression of GPX2 in Y-iPSCs (Figure S2A) induced the oxidation capacity of glutathione (Figure 1C). Conversely, short hairpin RNA (shRNA) knockdown of GPX2 in A-iPSCs (Figure S2B) led to a reduction in oxidation capacity of glutathione (Figure 1C). We also observed higher ROS scavenger activity upon GPX2 overexpression in Y-iPSCs (Figure 1D) and reduced ROS scavenger activity with shRNA knockdown of GPX2 in A-iPSCs (Figure 1D).

### GPX2 Regulates DNA Damage Response and Apoptosis in Pluripotent Stem Cells

**Figure 2:**
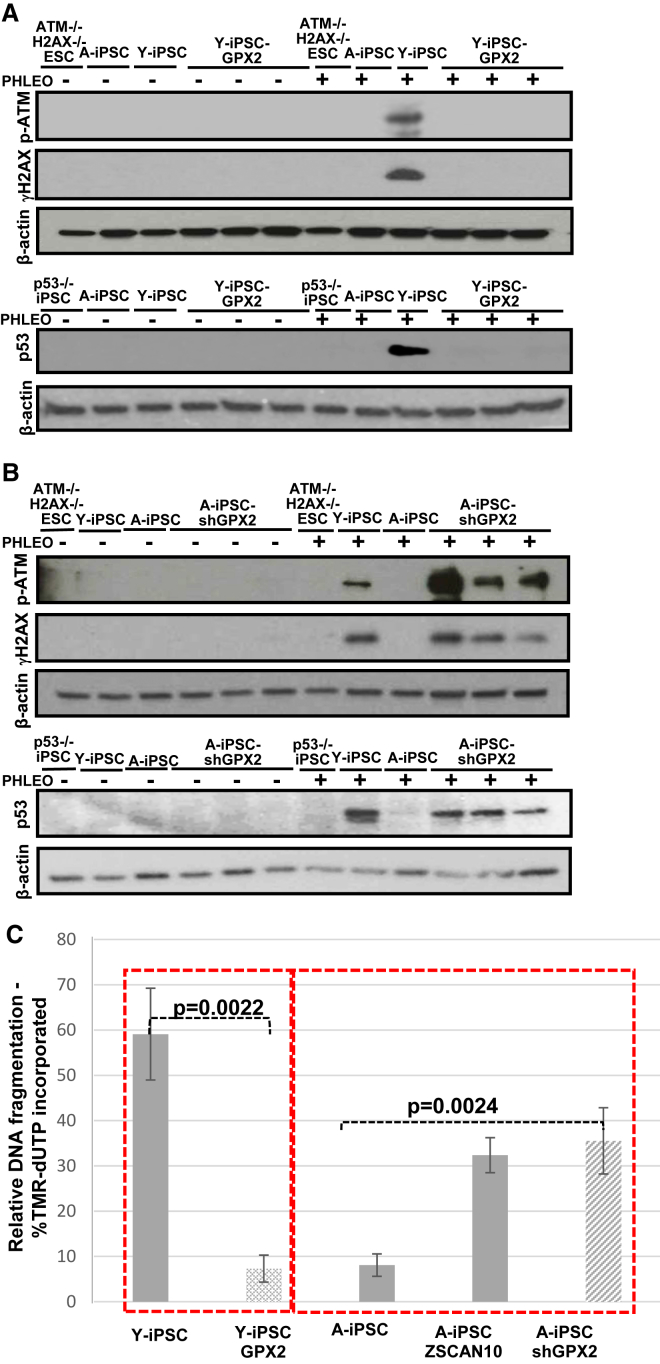
Poor DNA Damage Response and Apoptosis in A-iPSCs and Recovery by ZSCAN10 via Reduction of Excessively Activated GPX2 (A) Immunoblot of pATM/γH2AX/p53 showing an impaired DNA damage response after phleomycin treatment in A-iPSCs and three independent clones of Y-iPSCs with lentiviral expression of GPX2 cDNA (also refer to Figure S2A). (B) Immunoblot of pATM/γH2AX/p53 showing recovery of the DNA damage response after phleomycin treatment in three independent clones of A-iPSCs with GPX2 shRNA expression (also refer to Figure S2B). (C) Apoptosis detected by flow cytometry in Y-iPSCs with GPX2 overexpression and A-iPSCs with ZSCAN10 or GPX2 shRNA expression. We observed a lower apoptotic response (DNA fragmentation assay) 15 hr after the end of phleomycin treatment (2 hr, 30 μg/mL) in A-iPSCs and recovery with GPX2 downregulation in A-iPSCs to levels similar to that of A-iPSC-ZSCAN10. Transient expression of GPX2 in Y-iPSCs also reduces apoptotic response to the levels seen in A-iPSCs. Mean ± SD is plotted for multiple replicates (Y-iPSC = 6, Y-iPSC-GPX2 = 3, A-iPSC = 3, A-iPSC-ZSCAN10 = 3, A-iPSC-shGPX2 = 4). Statistical significance was determined by two-sided t test (also refer to Figure S4C) (three independent experiments).

These data suggest that the elevated glutathione activity and lower ROS levels in A-iPSCs are related to higher *Gpx2* expression, which leads to a homeostatic imbalance between ROS and glutathione that impairs the DNA damage response (Skamagki et al., 2017). Therefore, we investigated whether GPX2 overexpression is one of the main drivers of the increased glutathione and the blunted DNA damage response in A-iPSCs. We found that shRNA knockdown of GPX2 in A-iPSCs recovered the DNA damage response, as indicated by activation of the pATM, γH2AX, and p53 pathways (Figure 2B). Conversely, overexpression of GPX2 in Y-iPSCs blunted the DNA damage response (Figure 2A). GPX2 overexpression and lower ROS in Y-iPSCs was correlated with significantly reduced apoptosis (Figures 2C and S4C) (Franco and Cidlowski, 2009) and poor activation of the DNA damage response pathway (Guo et al., 2010, Sleigh, 1976). shRNA knockdown of GPX2 and higher ROS in A-iPSCs recovered the apoptosis rate equivalent to that of Y-iPSCs (Figures 2C and S4C) with reactivation of the DNA damage response pathway.

### ZSCAN10 Targets Exosome Subunits to Maintain ARE-Mediated *GPX2* RNA Reduction

**Figure 3:**
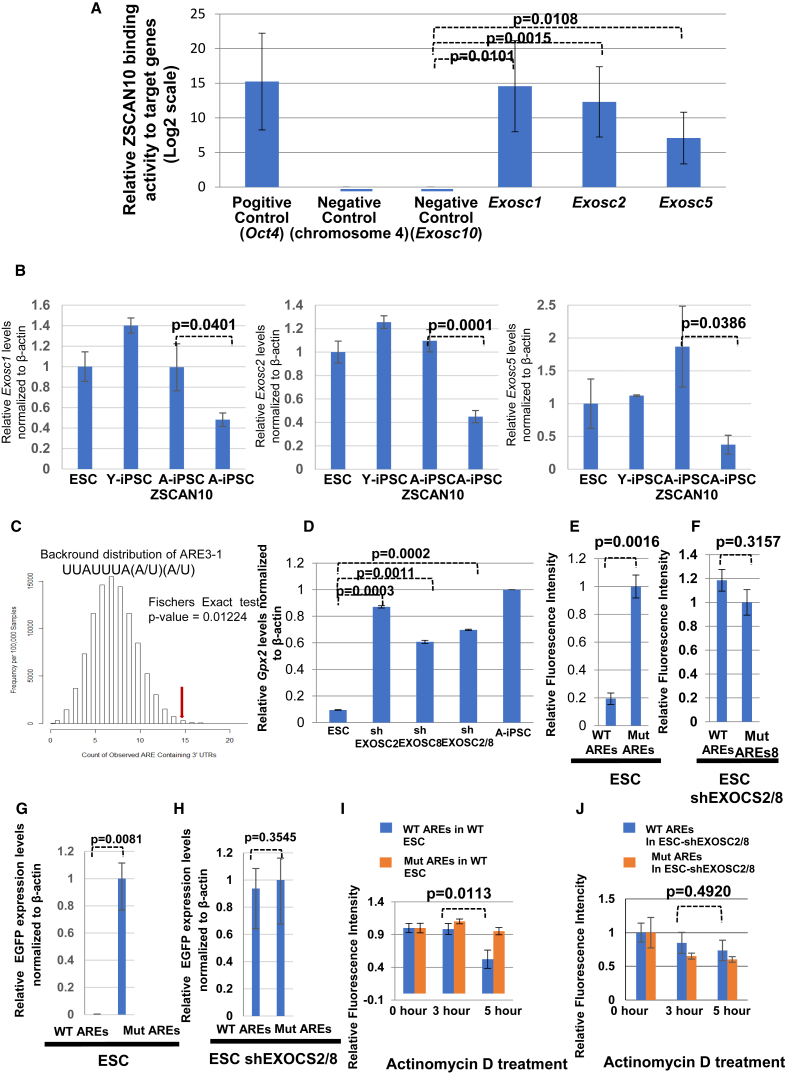
ZSCAN10 Targets the RNA Exosome Complex, which Influences *GPX2* Gene Expression (A) Confirmation of ZSCAN10 binding activity to the exosome subunits reported by Yu et al. (2009) using ChIP-qPCR. The graph shows the fold enrichment compared with a negative control and normalized by input. Error bars indicate the SEM of three replicates (n = 3). Statistical significance was determined by two-sided t test. The *Oct4* promoter region was used as a positive control and an 80 bp region on chromosome 4 was used as a negative control. The *Exosc10* region that did not show ZSCAN10 binding activity was used as an additional negative control. (B) Correlation between *Zscan10* expression levels and RNA exosome subunit expression. Error bars indicate SEM. Multiple independent replicates in each samples (A-iPSC = 5, A-iPSC-ZSCAN10 = 6, Y-iPSC = 3, ESC = 3). Statistical significance was determined by two-sided t test (also refer to Figure S4B). (C) Enrichment analysis for ARE3-1[UUAUUUA[AU][AU]] in the 3′ UTR (Zubiaga et al., 1995) of a set of genes/transcripts upregulated in A-iPSCs compared with ESCs/Y-iPSCs/A-iPSC-ZSCAN10. The comparison was performed against a control whole-genome transcript pool (Sharova et al., 2009). The vertical red line indicates overlap of 14 such transcripts out of 60 interrogated. The histogram represents a random probability distribution of overlap (p = 0.012; 100,000 permutations). Statistical significance was estimated by Fisher's exact test. (D) Gene expression analysis of *Gpx2* mRNA with shRNA knockdown of EXOSC2 and/or EXOSC8 by qPCR. The *Gpx2* mRNA level is low in ESCs, and shRNA knockdown of EXOSC2 and/or EXOSC8 significantly increases *Gpx2* expression. Mean ± SD is plotted for three replicates in each sample group from each condition (n = 3). Statistical significance was determined by two-sided t test. (E) Low expression in ESCs of a destabilized form of GFP reporter construct containing wild-type GPX2 AREs and recovery of expression after introduction of mutations in the GPX2 AREs (n = 3). (F) Recovery of wild-type GPX2 ARE reporter construct in ESCs expressing shEXOSC2/8. Mean ± SD is plotted for three replicates in each sample group from each condition (three independent experiments). Statistical significance was determined by two-sided t test (n = 3). (G and H) The expression level of the destabilized EGFP reporter was further confirmed using qPCR to measure the mRNA levels of EGFP under the control of wild-type (WT) and mutant (MUT) GPX2 ARE sequences in both ESCs (G) and ESCs expressing shRNA EXOSC2/8 (H). The RNA samples were collected 5 hr post actinomycin D treatment (0.3 μg/mL). Fold changes in EGFP under the control of WT-GPX2-ARE are shown relative to EGFP mRNA levels under the control of MUT-GPX2-ARE. Fold change is plotted for three replicates for each group from each condition (n = 3). Statistical significance was determined by two-sided t test. Error bars indicate SEM. (I) Measurement of GFP reporter expression rate for a reporter containing wild-type GPX2 AREs versus mutant GPX2 AREs. RNA degradation rate was monitored with inhibition of transcription by actinomycin D treatment. (J) Recovery of rapid reduction in expression of the wild-type GPX2 ARE GFP reporter in ESCs expressing shEXOSC2/8. Mean ± SD is plotted for three replicates in each sample group from each condition (n = 3) (three independent experiments). Statistical significance was determined by two-sided t test.

From a comparative gene expression analysis of ESCs, Y-iPSCs, A-iPSCs, and A-iPSCs expressing ZSCAN10, we observed that ZSCAN10 expression normalized *Gpx2* expression in A-iPSCs, and high GPX2 expression was associated with higher ROS scavenging activity and a blunted DNA damage response. That led us to hypothesize that the PSC-specific transcription factor ZSCAN10 directly binds to the *Gpx2* promoter (Yu et al., 2009), but could not find any binding activity near the *Gpx2* genomic region. However, we found that *Gpx2* contains four AREs, which are targeted by ARE-binding proteins to the RNA exosome complex. AREs were defined by measuring the decay rate of the RNA with the specific target consensus DNA sequence (Zubiaga et al., 1995). Therefore, we hypothesized that ZSCAN10 regulates *Gpx2* expression indirectly by controlling RNA exosome complex subunit gene expression. Chromatin immunoprecipitation (ChIP)-on-Chip data showed strong binding activity of ZSCAN10 to the promoters of multiple exosome complex subunits, including *Exosc1/2/5* (Yu et al., 2009). We generated a biotin-tagged ZSCAN10-tomato fluorescent protein reporter in ESCs and performed ZSCAN10 ChIP-qPCR analysis. We found that ZSCAN10 binds to multiple EXOSC subunits, including EXOSC1/2/5, but had no binding activity to *Exosc10* as a negative control, thus confirming the published ChIP-on-Chip data (Yu et al., 2009) (Figure 3A). qPCR analysis of *Exosc*1/2/5 expression in ESCs/Y-iPSCs/A-iPSCs/A-iPSC-ZSCAN10 showed that relatively higher levels of *Zscan10* expression in ESCs/Y-iPSCs were correlated with higher levels of *Exosc1/2/5* gene expression (Figures 3B and S4B), whereas the lower levels of ZSCAN10 in A-iPSCs were correlated with lower levels of *Exosc*1/2/5 gene expression (Figures 3B and S4B). Furthermore, overexpression of ZCAN10 in A-iPSCs led to restoration of *Exosc1/2/5* gene expression (Figures 3B and S4B), demonstrating a mechanistic link between ZSCAN10 and exosome subunit expression. ARE consensus sequences are well studied, and functional inhibitory ARE consensus sequences based on comparative RNA stability analysis have been identified (Zubiaga et al., 1995). We hypothesized that if A-iPSCs contain fewer core RNA exosome subunits (and thus fewer functional RNA exosome complexes), we would expect to see a slower turnover of ARE-containing transcripts. To test this hypothesis, we performed gene enrichment analysis of a gene set containing the minimal AU-rich motif targeted by the RNA exosome complex that effectively destabilizes mRNA (Sharova et al., 2009, Zubiaga et al., 1995) among a list of genes highly expressed in A-iPSCs (poor expression of ZSCAN10) and in ESCs, Y-iPSCs, and A-iPSC-ZSCAN10 (normal ZSCAN10 expression level; Figure 3C). We observed significant ARE-containing gene enrichment in A-iPSCs (p = 0.012) (Figure 3C), suggesting that loss of ZSCAN10-mediated RNA exosome complex subunit expression in A-iPSCs allows significant upregulation of ARE-containing RNAs (Table S1). *Gpx2* was one of the functional ARE-containing transcripts upregulated in A-iPSCs. As a control, we also performed the gene enrichment analysis with a subset of ARE-containing sequences that did not show functional effects in the previous report (Zubiaga et al., 1995), and confirmed no significant enrichment (Figure S2C).

### Modulation of the RNA Exosome Complex Influences *GPX2* Gene Expression

Among the gene list (Table S1), the *Gpx2* gene contains multiple ARE sequences, suggesting a mechanism by which ZSCAN10, via the RNA exosome complex, could regulate GPX2 and the redox status of iPSCs. To test this hypothesis, we could modify expression levels of EXOSC1/2/5 in A-iPSCs equivalent to the gene expression level of ESCs/Y-iPSCs. Thus, we knocked down the expression of individual RNA exosome complex subunits in ESCs and monitored the levels of *Gpx2* gene expression. Because the exosome complex requires assembly of multiple subunits, the knockdown of a single core complex subunit can affect its function (Boczonadi et al., 2014, Flynn et al., 2011, Pefanis et al., 2014). We chose to knock down EXOSC2 because it is the subunit that is downregulated to the greatest extent in A-iPSCs, and it is required for stabilization of the hexameric ring of RNase PH-domain subunits by interacting with EXOSC4 and EXOSC7 (van Dijk et al., 2007). We disrupted the exosome complex by shRNA knockdown of EXOSC2 in ESCs (Figure S3), which led to a dramatic increase in *Gpx2* gene expression (Figure 3D), similar to the expression level in A-iPSCs (Figure 3D). To monitor independent disruption of the exosome complex function by knockdown of an unrelated exosome subunit, we also knocked down EXOSC8 expression (Figure S3), which is known to upregulate ARE-containing RNAs (Boczonadi et al., 2014), but is not targeted by ZSCAN10 in PSCs. We observed that shRNA knockdown of EXOSC8 also enhances *Gpx2* gene expression (Figure 3D). In addition, shRNA knockdown of both EXOSC2 and EXOSC8 elevated *Gpx2* expression but did not show an additive effect (Figure 3D), confirming that the knockdown of a single exosome subunit is sufficient to disrupt RNA exosome complex function. The mechanistic link between the RNA exosome complex and *Gpx2* expression was further confirmed in ESCs by the poor expression of a GFP (a destabilized form of GFP) reporter construct containing wild-type GPX2 AREs and recovery of reporter expression after introduction of mutations in the GPX2 AREs (Figure 3E). Expression of the wild-type GPX2 ARE reporter in ESCs was also recovered with expression of EXOSC2/8 shRNA (Figure 3F). Inhibition of transcription by actinomycin D treatment led to a faster reduction in wild-type GPX2 ARE reporter expression than the mutant GPX2 ARE reporter expression (Figure 3I). Again, wild-type GPX2 ARE reporter expression could be rescued in ESCs expressing EXOSC2/8 shRNA (Figure 3J). The expression of wild-type and mutant GPX2 ARE GFP reporters in ESCs, with or without EXOSC2/8 shRNA, was confirmed by qPCR (Figures 3G and 3H).

### RNA Exosome Complex Controls GPX2 to Maintain Redox Homeostasis and DNA Damage Response

**Figure 4:**
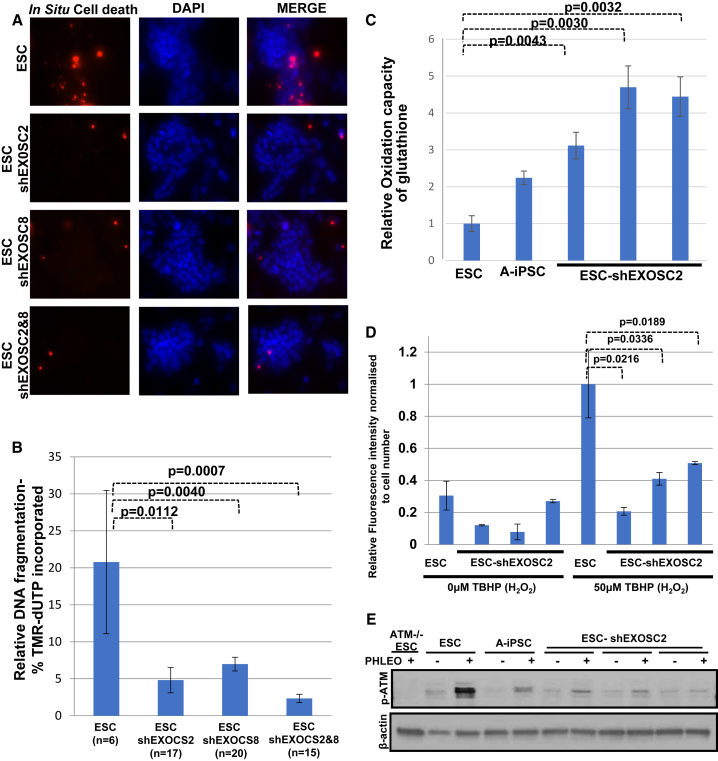
Recapitulation of poor Apoptosis, Imbalance of ROS/Glutathione Homeostasis, and Poor DNA Damage Response in ESCs by shRNA EXOSC2/8 Expression (A) *In situ* cell death assays of ESCs with or without shRNA for EXOSC2, EXOSC8, or both EXOSC2/EXOSC8 were performed 15 hr after the end of phleomycin treatment (2 hr, 30 μg/mL). ESCs with either EXOSC2 shRNA, EXOSC8 shRNA, or both shRNAs show fewer cells staining for cell death compared with ESCs. Nuclei are stained with DAPI. Scale bar indicates 100 μm. (B) Quantification by image analysis of the apoptotic response in the DNA fragmentation assay after phleomycin treatment. Error bars indicate the SEM of multiple replicates (ESC = 8, ESC-shEXOSC2 = 17, ESC-shEXOSC8 = 20, ESC-shEXOSC2 and 8 = 15). Statistical significance was determined by unpaired two-sided t test. (C) Quantification of the reduced form of glutathione in ESCs and ESCs with EXOSC2 shRNA. Mean ± SD is plotted for multiple replicates from each condition (ESC = 4, A-iPSC = 6, ESC-shEXOSC2 cl.1 = 3, ESC-shEXOSC2 cl.2 = 3, ESC-shEXOSC2 cl.3 = 3) (three independent experiments). Statistical significance was determined by two-sided t test (also refer to Figure S4D). (D) H_2_O_2_ scavenging activity in ESCs and ESCs with either EXOSC2, EXOSC8, or EXOSC2/EXOSC8 shRNA. Mean ± SD is plotted for three replicates in each sample group from each condition (n = 3) (three independent experiments). Statistical significance was determined by two-sided t test. (E) Immunoblot of pATM/γH2AX/p53 shows impaired DNA damage response after phleomycin treatment in ESCs and ESCs with either EXOSC2, EXOSC8, or EXOSC2/EXOSC8 shRNA.

Because *Gpx2* gene expression was increased by knockdown of RNA exosome complex subunit gene expression, we hypothesized that knockdown of exosome subunits by shRNA would also reduce the apoptotic response to exogenous stress (phleomycin treatment) in ESCs, similar to the effect of GPX2 overexpression in Y-iPSCs. Indeed, knockdown of EXOSC2 or EXOSC8 in ESCs significantly reduced the apoptotic response to stress (Figures 4A and 4B), with no significant additive effect of knocking down both EXOSC2 and EXOSC8 (Figures 4A and 4B). We further assessed the effect of EXOSC2 knockdown on the homeostatic imbalance between glutathione and ROS and the DNA damage response in ESCs. Similar to what was seen with overexpression of GPX2 in Y-iPSCs, knockdown of EXOSC2 in ESCs elevated the oxidation capacity of glutathione and reduced ROS scavenging activity (Figures 4C, 4D, and S4D). In addition, the DNA damage response was disrupted by EXOSC2 knockdown (Figure 4E), indicating that the RNA exosome complex has a significant role in regulating *Gpx2* expression levels to maintain the balance between glutathione and ROS and support a normal DNA damage response.

## Discussion

In summary, we found that a pluripotent transcription factor, ZSCAN10, binds near the transcription start sites of multiple RNA exosome complex subunits (*Exosc*1/2/5) in PSCs, as reported previously (Yu et al., 2009). Poor expression of ZSCAN10 in A-iPSCs led to poor exosome subunit expression and an increase in ARE-containing RNAs, including *Gpx2*. Elevated GPX2 in turn increases the reduced form of glutathione (active form), which scavenges ROS, blunts the DNA damage response, and reduces apoptosis. Expression of ZSCAN10 in A-iPSCs normalizes RNA exosome subunit expression, which reduces GPX2 and restores glutathione homeostasis and the DNA damage response. Taken together, these results provide mechanistic insights into the role of the RNA exosome regulatory pathway in PSCs, as well as the regulation of the RNA exosome complex itself by the pluripotent factor ZSCAN10 (Figure S1). Here, we report that the PSC-specific transcription factor ZSCAN10 can sustain the expression level of the RNA exosome complex subunits 1/2/5 in PSCs, and that knockdown of EXOSC2 and/or EXOSC8 causes similar effects in ESCs, with no significant additive effect of knocking down the combination. This suggests that each subunit is necessary for the function of the entire RNA exosome complex. However, certain exosome subunit mutations have been found to be more dominant and cause specific phenotypes. For example, mutations in *Exosc2* are specifically associated with a syndrome characterized by retinitis pigmentosa, progressive hearing loss, premature aging, short stature, mild intellectual disability, and distinctive gestalt (Di Donato et al., 2016). This may indicate that *Exosc2* mutations have a more deleterious effect than mutations in other exosome subunits. Another possibility is that each exosome subunit mutation causes a selective advantage to target specific ARE-containing RNAs through alterations in the overall exosome complex. Additional biological roles of the specific RNA exosome complex subunits *Exosc*1/2/5, and their regulation by ZSCAN10 in PSCs, remain to be studied. With regard to ZSCAN10 as a regulator of the exosome complex, it would be interesting to identify the upstream factors that regulate the differential activation of ZSCAN10 in Y-iPSCs and A-iPSCs. The mechanism may involve differential epigenetic regulation, given that the cell lines used here have the same genetic background. While epigenetic regulation can be altered by aging, it may be challenging to directly connect differences in ZSCAN10 activation to differences in somatic cell epigenetic memory, because ZSCAN10 is only expressed in PSCs. As increasing the passage number did not recover ZSCAN10 expression in A-iPSCs, we are currently investigating epigenetic mechanisms upstream of ZSCAN10 that may regulate its expression. An indirect influence of the accumulation of epigenetic modifications with aging might lead to poor activation of ZSCAN10 in A-iPSCs versus Y-iPSCs. Once the mechanism of ZSCAN10 activation has been deciphered, the next step would be to identify small molecules that can directly affect these pathways and explore their potential for improving A-iPSC quality. ZSCAN10-targeted genes have been reported by Yu et al. (2009) using ChIP-on-Chip analysis. Genes that are directly or indirectly regulated by ZSCAN10 need to be validated and their specific functional roles identified.

## Experimental Procedures

ESCs and iPSCs were cultured in ESC medium containing 20% fetal bovine serum and 1,000 U/mL of leukemia inhibitory factor. For reprogramming of somatic cells, retroviruses expressing OCT4, SOX2, KLF4, and MYC were introduced. Information regarding cell lines, antibodies, plasmids, and drugs used in this study, as well as detailed protocols for reprogramming and cell culture, overexpression and knockdown, gene expression analysis (qPCR), immunofluorescence staining and analysis, immunoblot analysis, retrovirus and lentivirus production, drug treatments, glutathione detection assay, and H_2_O_2_ ROS assay are provided in the Supplemental Experimental Procedures. The animal study is compliant with all relevant ethical regulations regarding animal research by an Institutional Animal Care and Use Committee (IACUC approval number 11-10-023).

## Author Contributions

M.S., R.Z., and K.K. conceived the experimental plan. M.S., A.A., and Z.L. performed the experiments. C.Z., C.A.R., Q.M., J.W., and H.L. performed the computational analysis. M.S., C.Z., C.A.R., Z.L., Q.M., U.B., J.W., R.Z., H.L., and K.K. wrote the manuscript.

## Accession Numbers

Microarray data that support the findings of this study have been deposited in the Gene Expression Omnibus under accession code GEO: GSE85365.

## Supplemental Information

Document S1. Supplemental Experimental Procedures and Figures S1–S4

Table S1. Upregulated genes in A-iPSC compared to A-iPSC-ZSCAN10 (more than 3-fold), with ARE information

Document S2. Article plus Supplemental Information
