# Supplementary material for: RNA Exosome Complex-Mediated Control of Redox Status in Pluripotent Stem Cells
Source: Stem Cell Reports. 2017 Oct 10;9(4):1053–61. doi: 10.1016/j.stemcr.2017.08.024 (PMC5639470; doi:10.1016/j.stemcr.2017.08.024)
Supplement: Document S1. Supplemental Experimental Procedures and Figures S1–S4 [file mmc1.pdf]

Stem Cell Reports, Volume *9*

Supplemental Information

RNA Exosome Complex-Mediated Control of Redox Status in Pluripotent Stem Cells

Maria Skamagki, Cheng Zhang, Christian A. Ross, Aparna Ananthanarayanan, Zhong Liu, Quanhua Mu, Uttiya Basu, Jiguang Wang, Rui Zhao, Hu Li, and Kitai Kim

Figure. S1. Schematic regulatory model of the homeostatic balance between ROS and glutathione by the RNA exosome complex. (Related to Figures 1-4) We previously found that A-iPSC show transcriptional alterations, including poor expression of the pluripotent factor ZSCAN10, compared to Y-iPSC (Skamagki et al. 2017). In this study, we report that the pluripotent factor ZSCAN10 binds directly to the promoters of multiple components of the RNA exosome complex and stimulates their expression. Concurrently, we found that the glutathione peroxidase 2 (GPX2) RNA transcript contains AREs targeted by the RNA exosome complex. GPX2 increases the reduced form of glutathione (GSH: active form) from oxidized glutathione (Chu et al. 2004). Glutathione is a scavenger metabolite for reactive oxygen species (ROS), and the homeostatic balance between glutathione and ROS is important to maintain the DNA damage response signalling pathway and maintain genomic stability (Sleigh 1976; Franco and Cidlowski 2009). Loss of the homeostatic balance with lower glutathione causes an excess of ROS, which directly damages DNA. Conversely, because ROS is an important cellular signal of stress that induces the DNA damage response, loss of the homeostatic balance with excessive glutathione depletes ROS and leads to a defective DNA damage response. This increases cell exposure to additional genotoxic stresses, and leads to accumulation of mutations (Sleigh 1976; Guo et al. 2010; Harris et al. 2015). We utilized A-iPSC to test the hypothesis that ZSCAN10 indirectly regulates GPX2 expression in pluripotent stem cells *via* interaction with the RNA exosome complex to maintain the ROS-glutathione balance in pluripotent stem cells. In our proposed model, poor expression of ZSCAN10 in A-iPSC reduces RNA exosome complex subunit expression and consequently limits ARE-mediated RNA degradation, including that of GPX2 transcripts. Consequently, the elevated levels of GPX2 in A-iPSC increase the active form of glutathione, causing a homeostatic imbalance between ROS and glutathione.

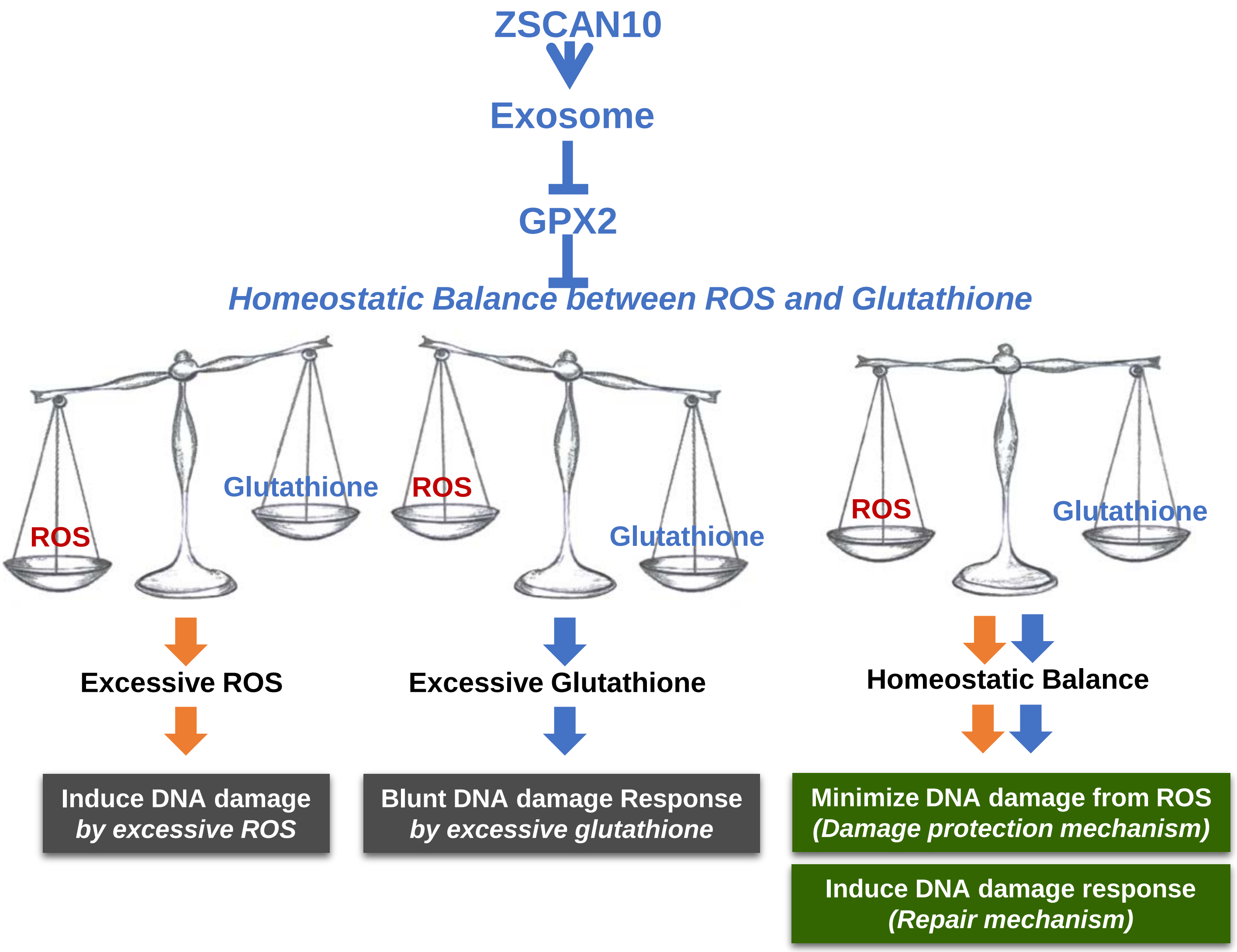

Table S1. Gene enrichment analysis of a gene set containing the minimal AU-rich motif targeted by the RNA exosome complex that effectively destabilizes mRNA transcripts. Upregulated genes in A-iPSC compared to A-iPSC-ZSCAN10 (more than 3-fold), with ARE information (Related **to Figure 3C**; also refer to the independent Excel files). We performed gene enrichment analysis of a gene set containing the minimal AU-rich motif targeted by the RNA exosome complex that effectively destabilizes mRNA transcripts. The gene set included genes that are highly expressed in A-iPSC (poor expression of ZSCAN10) compared to ESC, Y-iPSC, and A-iPSC-ZSCAN10 (normal ZSCAN10 expression level; **Figure 3C**). The comparison was performed against a control whole genome transcript pool (Sharova et al. 2009). We observed significant ARE-containing gene enrichment in A-iPSC (p=0.012) (**Figure 3C**), suggesting that loss of ZSCAN10-mediated RNA exosome complex subunit expression in A-iPSC allows significant upregulation of ARE-containing RNAs (**Table S1**).

Related to Figure 3C Refer the independent Excel files.

Figure S2. Expression level of *Gpx2* after shRNA and cDNA expression and gene enrichment analysis. (Related to Figures 1, 2 & 3C) (A) *Gpx2* expression levels in Y-iPSC after overexpression of GPX2. (B) Expression levels of *Gpx2* after shRNA knockdown in A-iPSC. Mean ± standard deviation is plotted for three independent replicates for *Gpx2* overexpression and shRNA knockdown in each sample group from each condition (n=3). Statistical significance was determined by two-sided t-test. (C) Gene enrichment analysis was performed with ARE1[AUUUA] and/or ARE2-1[UUAUUUAUU] in a set of genes/transcripts that were upregulated in A-iPSC compared to ESC/Y-iPSC/A-iPSC-ZSCAN10. The comparison was performed against a control whole genome transcript pool (Sharova et al. 2009). Vertical red line indicates overlap of 41 such transcripts out of 60 interrogated. The histogram represents a random probability distribution of overlap (100,000 permutations). The figure demonstrates a mathematical point about sampling distributions: that the likelihood of any given transcript to have an ARE is x, so the odds of us seeing this many in a sample based on just random chance is Y. Based on the background of ARE1-2s in the population (13,611/19,977 transcripts), we observed more than half of transcripts have an ARE (41 out of 60 transcripts); therefore, there is no significant enrichment.

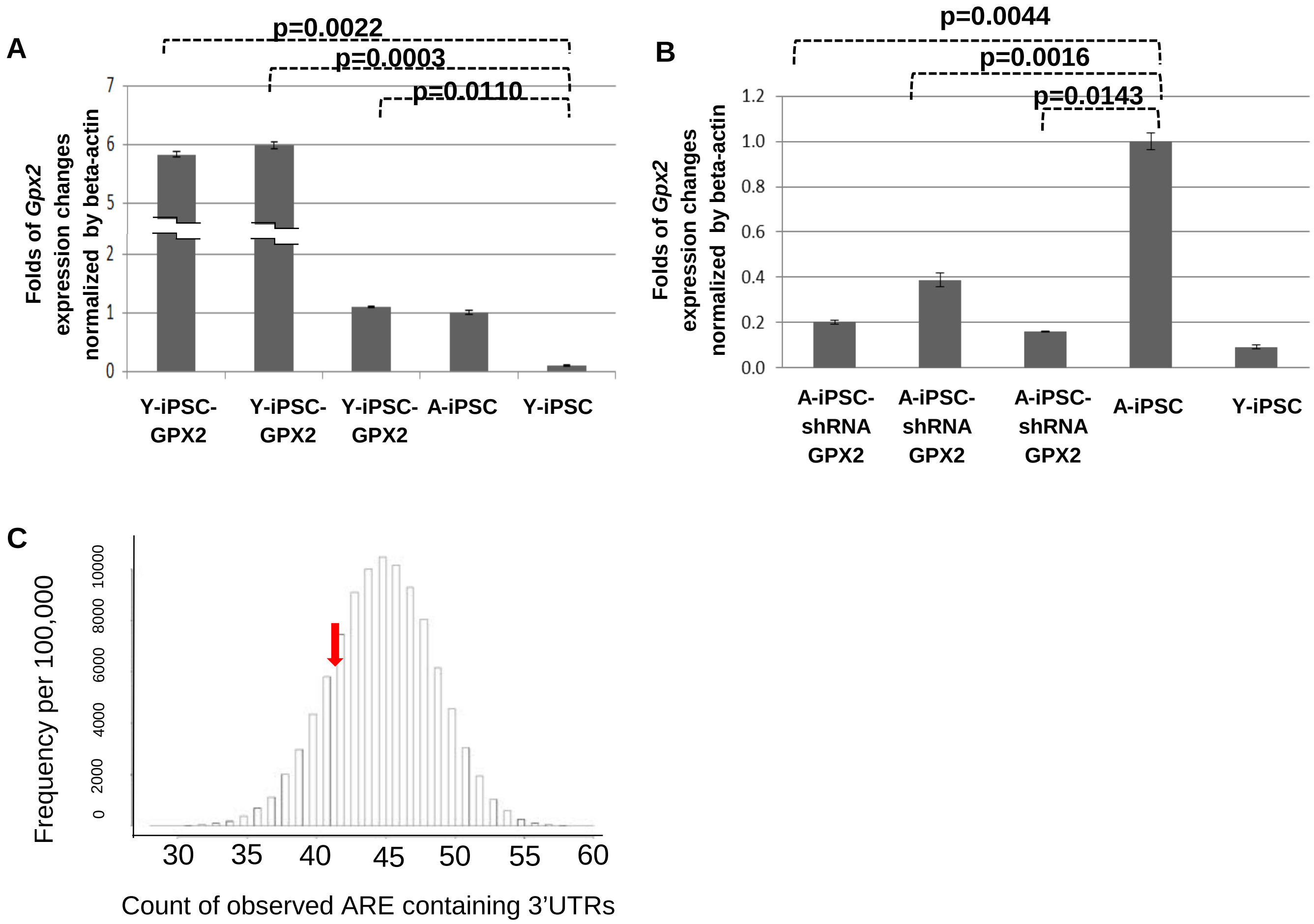

Figure S3. Expression level of *Exosc2* and *Exosc8* after shRNA knockdown in ESC. (Related to Figures 3 and 4) (A) Expression levels of *Exosc2* after shRNA knockdown of EXOSC2 or EXOSC2/8 in ESC. (B) Expression levels of *Exosc8* after shRNA knockdown of EXOSC8 or EXOSC2/8 in ESC. Mean ± standard deviation is plotted for three replicates for ESC shEXOSC2 or shEXOSC2/8 cells (n=3), and four replicates for ESC and A-iPSC in each sample group (n=4). Statistical significance was determined by two-sided t-test.

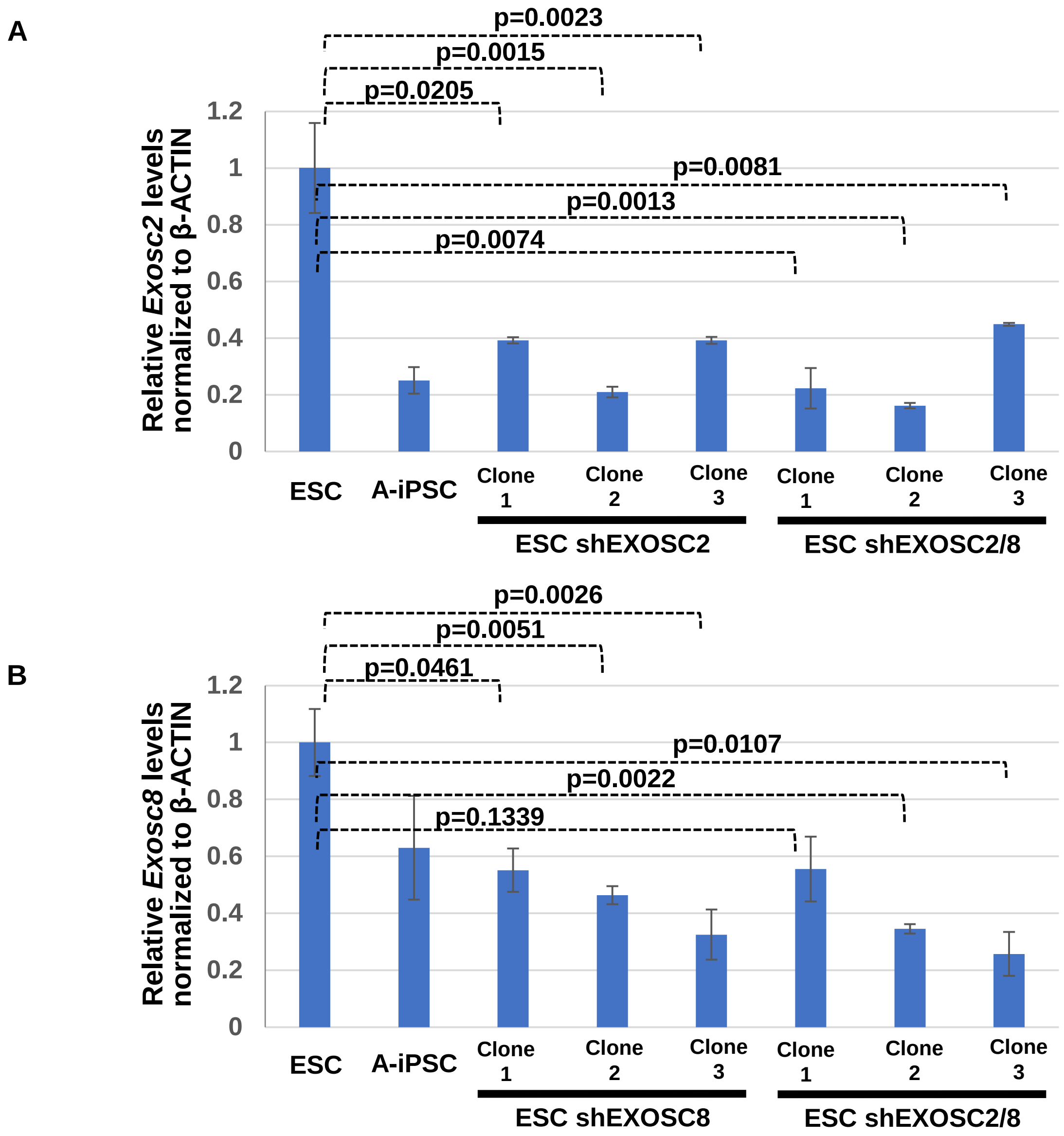

Figure S4. Clonal variability (Related to Figures 1-4). (A) Individual *Gpx2* values from microarray gene expression analysis (refer to Figure 1A). (B) Expression levels of *Exosc*1, 2, and 5. The scatter plot shows variability within each group. Colors indicate data points from the same clone. Error bars indicate standard error of the mean (refer to Figure 3B). (C) Apoptosis detected by flow cytometry in Y-iPSC with GPX2 overexpression and A-iPSC with ZSCAN10 or GPX2 shRNA expression. The scatter plot shows variability within each group. Colors indicate data points from the same clone (refer to Figure 2C). Mean ± standard deviation is plotted for multiple replicates. (D) Quantification of the reduced form of glutathione (GSH). The scatter plot shows the variability within a group. Colors indicate data points from the same clone. Mean ± standard deviation is plotted for multiple replicates (refer to Figure 4C).

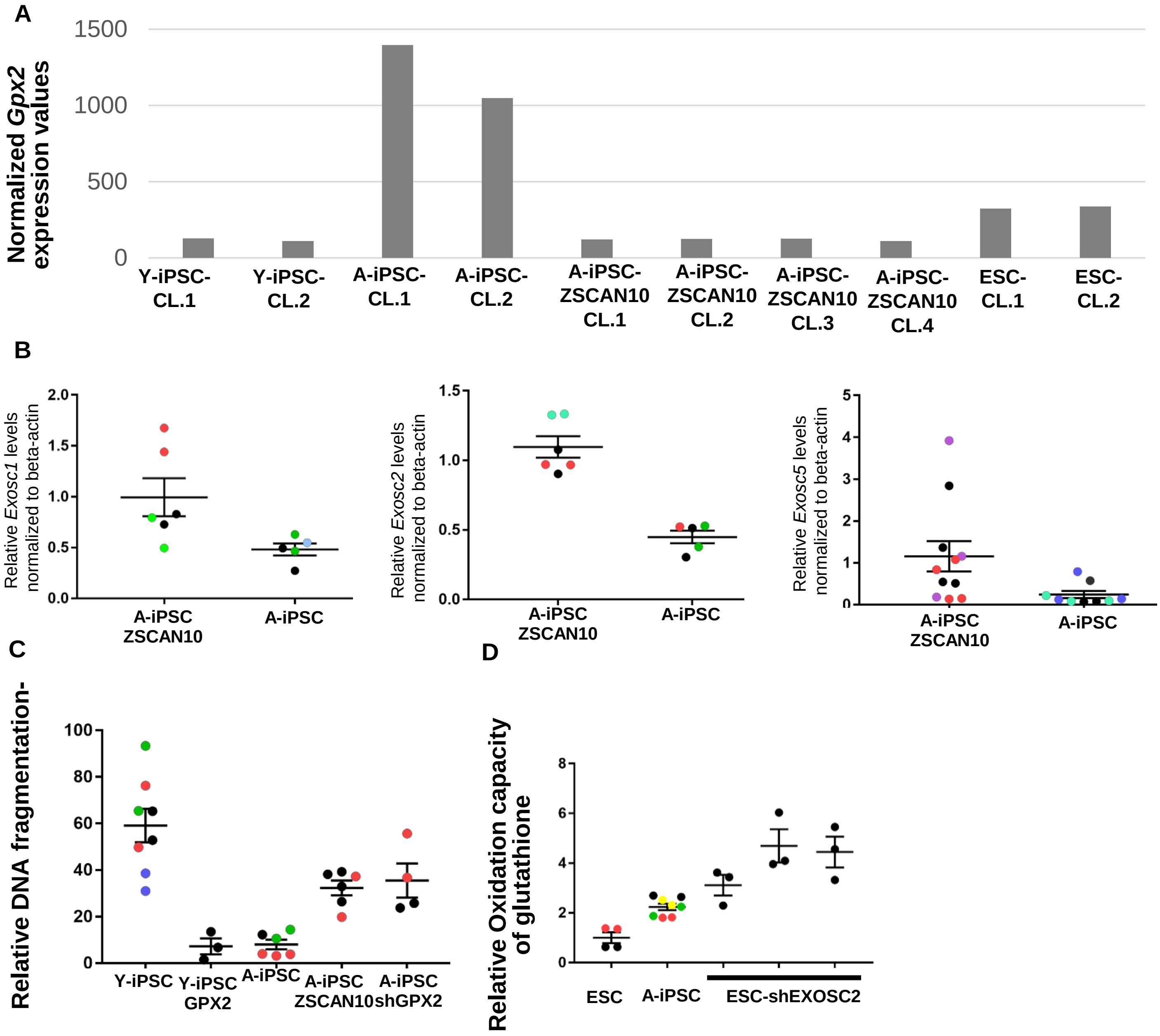

METHODS

Cell culture

Mouse ESC and iPSC were cultured in ESC media containing 20 % FBS and 1,000 U/ml of LIF (ESGRO® Leukemia Inhibitory Factor [LIF], 1 million units/1 mL). Mouse ESC were generated and their pluripotency was tested as reported in our previous publication (Kim et al., 2010). Doxycycline-inducible ZSCAN10 was induced in media supplemented with 2 µg/ml doxycycline (MP Biomedicals, doxycycline hyclate).

Generation of Y-iPSC, A-iPSC, A-iPSC-ZSCAN10

Fibroblasts were collected from B6CBAF1 mouse E17.5 embryonic skin, 5-day-old tail tip skin, and 1.5-year-old tail tip skin. Fibroblasts were not cultured for more than 3 passages to limit extensive *in vitro* culture. 10^6 fibroblasts were infected with retrovirus generated from pMX-mOCT4, pMX-mSOX2, pMX-mKLF4 (Koh et al., 2002; Moran et al., 2006) in 6-well dishes with 0.5 ml of each viral supernatant (total 2 ml per well) and spun at 2500 rpm at RT for 90 min (BenchTop Centrifuge, BeckmanCoulter, Allegra-6R). For the generation of A-iPSC-ZSCAN10 , the procedure was identical, but in addition to the four reprogramming factors, we added a doxycycline-inducible system to overexpress ZSCAN10 . This system consisted of two lentiviruses generated from a plentiRZ–ZSCAN10/plentiRZ-GPX2 and a plenti-RTTA vector (Kim et al., 2011). All cells infected with the reprogramming factors and those with additional ZSCAN10 were plated on irradiated CF-1 mouse embryonic feeder cells in a 10-cm tissue culture dish in ESC media containing 20% FBS and 1,000 U/ml of LIF. Media were changed on day 2 and doxycycline addition started on day 3 to induce ZSCAN10 overexpression. Floating cells were collected by media centrifugation and returned to culture during media changes. On day 4, cultured cells were trypsinized and replated onto four 10-cm dishes pre-coated with gelatin (0.1%) and irradiated mouse embryonic fibroblasts (MEFs) in ESC maintenance media. Media were changed daily until ESC-like colonies were observed. The reprogrammed colonies were tested for pluripotency based on teratoma formation, alkaline phosphatase staining, SSEA-1 and NANOG staining, and OCT4 expression levels.

Generation of ZSCAN10 tomato-fluorescence protein reporter by CRISPR targeting

The 5′ end of the ZSCAN10 starting codon region in ESC was targeted to insert the Tomato fluorescent protein using the CRISPR genomic targeting tool. Briefly, the left and right arms of mZSCAN10 were cloned into a plasmid PCR2.1 IRES TOMATO P2A flagbio (Invitrogen) using the following primer sets

LEFT-ZSCAN10-nhe-F:	attgctagcGAGGACTACTTGTGGAAGTCAGTG
LEFT-ZSCAN10-bamh-R:	attggatccggatccttgggagaattcaggg
RIGHT-ZSCAN10-NOT-F:	attGCGGCCGCatgctggcggaaccagtccc
RIGHT-ZSCAN10-ASC-R:	attGGCGCGCCacagacagattggacagccaggac

The resulting plasmid, along with gRNA:
CACCgATACTGCGTTAAGATCTGAC
aaacGTCAGATCTTAACGCAGTATc
CACCgTTTAGCTCCACAGGTGCAGG
aaacCCTGCACCTGTGGAGCTAAAc

The primer sets and Cas9 were transfected into ESC, and individual clones were screened for integration by PCR and DNA sequencing.

Retrovirus generation

293T cells were seeded overnight at 5×10^6 cells per 150-mm dish with DMEM supplemented with 10% FBS and penicillin/streptomycin. Retrovirus was generated using pMX-mOCT4, pMX-mSOX2, pMX-mKLF4, and pEYK-mMYC as described previously (Koh et al., 2002; Takahashi and Yamanaka, 2006). The cells were transfected with calcium phosphate as previously described (Takahashi and Yamanaka, 2006). Media were replaced with fresh DMEM twice, 18 hours after transfection. Approximately 48 hours after transfection, medium containing the retrovirus was collected and the cellular debris was removed with centrifugation. The supernatant was filtered through a 0.45-µm filter, and the retrovirus was pelleted with ultracentrifugation at 33,000 rpm in a

45Ti rotor (Beckman) for 90 min at 4°C. The retroviral particles were resuspended in ESC medium and stored at -80°C.

Lentivirus production

293T cells were seeded overnight at 5×10^6 cells per 150-mm dish with DMEM supplemented with 10% FBS and penicillin/streptomycin. The cells were transfected with plentiRZ-ZSCAN10 (mouse and human), plenti-RTTA, or plenti-GPX2, with calcium phosphate cell transfection, as previously described (Kim et al., 2011). The ZSCAN10 cDNA was clone MmCD00295052 in the pENTR223.1 backbone and the GPX2 cDNA was clone MmCD00317538 in the pCMV SPORT6 backbone from the Harvard Plasmids core (http://plasmid.med.harvard.edu/PLASMID/Home.jsp). The cDNA for mouse ZSCAN10 was subcloned into a plentiRZ vector. At 48 hours after transfection, the medium containing the lentivirus was collected and the cellular debris was removed with centrifugation. The supernatant was filtered through a 0.45-μm filter, and the lentivirus was pelleted with ultracentrifugation at 33,000 rpm in a 45Ti rotor (Beckman) for 90 min at 4°C. The lentivirus particles were resuspended in DMEM medium and stored at -80°C.

Quantitative real time-PCR (Q-PCR) analysis

The expression levels of various genes (ZSCAN10, OCT4, GPX2, EXOSC1/2/5 and β-actin) were quantified by Q-PCR. Total RNA (1 μg) was reverse transcribed in a volume of 20 μl using the M-MuLV Reverse Transcriptase system (New England Biolabs), and the resulting cDNA was diluted into a total volume of 200 μl. 10 μl of this synthesized cDNA solution was used for analysis. For pluripotency genes, each reaction was performed in a 25 μl volume using the Power SYBR Green PCR Mastermix (Applied Biosystems). The conditions were programmed as follows: initial denaturation at 95°C for 10 min followed by 40 cycles of 30 sec at 95°C, 1 min at 55°C, and 1 min at 72°C; then 1 min at 95°C, 30 s at 55°C, and 30 sec at 95°C. All of the samples were duplicated, and the PCR reaction was performed using a Mx3005P reader (Stratagene), which can detect the amount of synthesized signals during each PCR cycle. The relative amounts of the mRNAs were determined using the MxPro program (Stratagene). The amount of PCR product was normalized to a percentage of the expression level of β-actin. The PCR products of OCT4, ZSCAN10, GPX2, EXOSC1/2/5, and β-actin were also evaluated on 1.2% agarose gels after staining with ethidium bromide. The primers used to amplify the cDNA were the following:

The primers used to amplify the cDNA were the following:
OCT4-For: GGCTCTCCCATGCATTCAA and
OCT4-Rev: TTTAACCCCAAAGCTCCAGG
ZSCAN10-For: GGCTCAGAGGAATGCGTTAG and
ZSCAN10-Rev: CATCTACAGGCCCACCAGTT
GPX2-For: GTGCTGATTGAGAATGTGGC and
GPX2-Rev: AGGATGCTCGTTCTGCCCA
β-ACTIN-For: TCGTGGGTGACATCAAAGAGA and
β-ACTIN-Rev: GAACCGCTCGTTGCCAATAGT,
EXOSC2-For: CCCCAAGGAGCATCTGACAA and
EXOSC2-Rev: CCAACCCACCATTACCTCCC
EXOSC1-For: ATGGGTTGGTGATGGGCATAG and
EXOSC1-Rev: CCCATGCTGTCACTATTGGGT
EXOSC5-For: CCGATTCTACCGGGAATCACT and
EXOSC5-Rev: CTACATGGGCACAGACAGAGG

ChIP-Q-PCR

ChIP was performed according to the published protocol (Tian et al., 2012). Immunoprecipitation was performed with streptavidin beads (M280, Invitrogen). We used Q-PCR to analyze the EXOSC DNA fragments in the immunoprecipitated samples. Results are presented as fold enrichment against the negative control (log2 value), calculated by real-time Q-PCR to quantify the abundance of the DNA fragment of interest added to the ChIP reaction, with respect to the abundance of the DNA fragment found in the final immunoprecipitate. The primers used were GGTTCTTGCTTCCCATCCGA and GAGTTGGGGCTGAAAATGCG for the EXOSC1 binding region, AGCTCGCAAACCTCTCAGTG and CCCCCAAATCCTCACGTACC for the EXOSC2 binding region, GTGAGCAAAACCTGCTGTCC and AACTTGGTGAGGCTCGTACC for the EXOSC5 binding region, and GGAAACGGGTCTGAGGCTAC and CCTCGGGAGAGGTAGTGACA

for the EXOSC10 region. The ZSCAN10 binding site on the EXOSC were estimated based on published ChIP-on-Chip data (Yu et al., 2009). An Oct4 promoter region was used as a positive control (AGGAAAGAGGCCCCGGCCTT and CCAGAGAAGTGCTGGCTCTGCG) and an 80-bp genomic region on chromosome 4 was used as a negative control (GGTGGGTTACACCTCATCGG and TAACAGCACTTGTCAGGCGA).

Drug treatments

Phleomycin (Sigma) was added at 30 µg/ml for 2 hours. Cells were processed for analysis 30 min after phleomycin treatment unless indicated otherwise. After a 30-min recovery in ESC media, the cells were collected and processed for the immunoblot experiments. In the DNA fragmentation assay, the cells were given 15 hours to recover.

DNA fragmentation analysis

DNA fragmentation was measured using an *in situ* cell death assay kit (Roche) for visualization of DNA strand breaks by labelling the free 3′-OH termini with modified nucleotides (e.g., biotin-dUTP, DIG-dUTP, fluorescein-dUTP) in an enzymatic reaction. iPSC cells (1 x 10^5 cells) were treated with phleomycin (30 µg/ml) for 2 hours. Samples were collected as control or treated for analysis 15 hours after phleomycin treatment. Additionally, cells were treated with DNAase I recombinant (Roche) (10 min, 3 U/ml, at 15°C to 25°C) to induce DNA strand breaks, as a positive control for apoptosis. Medium containing floating cells and attached cells was centrifuged (1000 g, 5 min) and collected. Cells were processed for flow cytometry analysis or microscopy.

Immunoblot analysis

Treated and untreated cells (1 x 10^5 cells) were collected 30 min after the 2-hour phleomycin treatment (30 µg/ml). To harvest protein, 100-200 mL RIPA buffer (50 mM Tris- HCl [pH 7.4], 150 mM NaCl, 1% NP40, 0.25% Na-deoxycholate, 1 mM PMSF, protease inhibitor cocktail, and phosphatase inhibitor cocktail) was added to floating cell pellets and the remaining adherent cells. The samples were incubated on ice (10 min) and centrifuged (14,000 g, 10 min, 4°C). Protein concentrations were determined using a BCA protein assay kit (Pierce). Samples were adjusted to the same concentration with RIPA buffer (3000 µg/ml) and were combined with Laemmli Sample Buffer (BioRad) and β-Mercaptoethanol (Sigma) then heated at 95°C for 5 min and loaded onto a 4-15% Mini Protean TGX SDS-PAGE gel (BioRad). Samples on the SDS-PAGE gel were transferred to a 0.2-mm PVDF membrane at 100 V for 1 h, using a wet electro-transfer method (0.2 M glycine, 25 mM Tris, and 20% methanol). The membrane was blocked with 5% BSA in PBS-T (1 h at 4°C), followed by incubation with primary antibodies anti–phospho-ATM (Pierce, MA1-2020) (1:1000), anti-H2AX (Millipore, 05-636) (1:1000), anti-p53 (Leica Biosystems, P53-CM5P) (1:1000), or anti-beta actin (Cell Signaling, #4967) (1:5000) in blocking solution (5% BSA in phosphate-buffered saline containing Tween-20 [1:1000] PBS-T, overnight at 4°C). After primary antibody incubation, membranes were washed three times in PBS-T) prior to addition of secondary antibody labelled with peroxidase. Secondary antibodies were from Cell Signaling (1:10,000). ATM/H2AX negative ESC controls was imported from Xie's laboratory (Rass et al., 2013).

H_2O_2 reactive oxygen species (ROS) assay (DCFDA assay)

H_2O_2 scavenging activity was measured using a cellular reactive oxygen species assay kit (Abcam, ab113851). ESC/iPSC were labelled with 20 µM DCFDA (2',7'-dichlorofluorescein diacetate; a fluorogenic dye that measures hydroxyl, peroxyl, and other ROS activity within the cell), and cultured for 3 h with 50 µM TBHP (tert-butyl hydrogen peroxide; stable chemical form of H_2O_2). Cells were then analysed on a fluorescent plate reader. Mean ± standard deviation is plotted for four replicates from each condition.

Glutathione detection assay

Feeder-free cells were cultured on Matrigel-coated tissue culture plates in MEF-conditioned ESC-media. On day 3, the cells were washed in PBS and scraped and pelleted by centrifugation. Subsequent steps were performed using a Glutathione Fluorometric Assay Kit (cat# K264-100, Biovision Inc.) according to the manufacturer's manual. Briefly, cell pellets were homogenized in ice

cold glutathione assay buffer, preserved in perchloric acid, and centrifuged. Supernatants were neutralized with potassium hydroxide. After centrifugation, the supernatant was either used to detect reduced glutathione (GSH), or total glutathione was measured by reducing oxidized glutathione (GSSG) to GSH before measurement. For measuring GSSG concentrations specifically, existing GSH was quenched before reducing agent was applied. OPA (o-phtalaldehyde) probe, which reacts with GSH and emits fluorescence, was added to samples, and signal was acquired at Ex/Em=340 nm/420 nm on a Varioscan Flash by Thermo Scientific. Oxidation capacity of glutathione was determined by the quantity of total glutathione (GSH+GSSG).

Generation of A-iPSC-shGPX2, Y-iPSC-GPX2, ESC-shEXOSC2, ESC-shEXOSC8, and ESC-shEXOSC2&8

A-iPSC were infected post-reprogramming with a set of shRNA viruses for GPX2 (6 GIPZ Lentiviral shRNA vectors from Thermo Scientific: RMM4532-EG14776). Clones were selected with puromycin, and the levels of down-regulation were measured by Q-PCR. ESCs were infected with a set of shRNA viruses for EXOSC2 and/or EXOSC8 (2 GIPZ Lentiviral shRNA vectors for EXOSC2 from GE DHARMACON: RMM4431-200370629, RMM4431-200332733 and 3 GIPZ Lentiviral shRNA vectors for EXOSC8 from GE DHARMACON: RMM4532-EG69639). Clones were selected with puromycin. Y-iPSC were infected with a lentivirus carrying the GPX2 cDNA post-reprogramming (Harvard Plasmid Core (http://plasmid.med.harvard.edu/PLASMID/Home.jsp). The infected clones were assessed for GPX2 expression levels by Q-PCR.

GPX2 ARE reporter constructs

207 bp of a DNA fragment containing 2 AREs was amplified by PCR using mutated and wild type primer sets.

Wild type ARE primers:
attgctagcTATTATTTAAAGGCTTGTTTCTAATC
atgaattcTTAAATCATAAAGAGAAACAGAATC

Mutant ARE primers:
attgctagcTctTcTTcAAGGCTTGTTTCTAATC
atgaattcTTcAcTCATAAAGAGAAACAGAATC

In addition, NheI restriction enzyme sites were added at the 5′ end of the forward primer sets, and cloned into pGEMTeasy vector (Promega). The NheI and EcoR1 digested DNA insert was cloned into NheI and EcoRI sites of pDeEGFP-N1 (Clontech), which encodes a rapidly degraded form of GFP (half life: 2 hours). The resulting plasmids were transfected by electroporation to obtain GFP-positive ESC and control ESC-shEXOSC2. RNA stability was measured after addition of 0.2 µg/ml of actinomycin D (Casse et al., 1999) at different time points by fluorescence signal and Q-PCR of the transcript.

Electroporation of constructs

Briefly, $6x10^6$ of ESC or ESC shEXOSC2&8 were trypsinized, washed twice with PBS, and then diluted in 500 µl of EMBRYOMAX electroporation buffer (Millipore cat#ES-003-D). 40 µg of DNA of each GPX2 ARE expression construct (wild type or mutant) was added in each cell line and the mixture of cells and DNA was transferred to a Gene Pulser Cuvette (Biorad, cat# 165-2088). An exponential electroporation protocol was followed using a GENE PULSER XCELL (Biorad) with the following settings: 220 voltage (V), 950 capacitance (µF), 1000 resistance (Ω), 4 mm cuvette. The cells were then plated in a 10-mL dish with feeder MEF and allowed to recover overnight. The cells were analyzed two days later by flow cytometry. Approximately 40% of the cells were expressing the GFP, and the percentage was similar for both constructs.

Gene expression analysis

Illumina genome-wide gene expression arrays (MouseRef-8 v2.0 MouseRef-8 v2.0 BeadChip) were used for mRNA expression analysis.

Statistics and reproducibility

Data were analysed using GraphPad Prism 5.0.1 software (GraphPad Software) and/or Excel software (Microsoft Office). Data are presented as mean with s.e.m. or s.d. (as indicated in the figure legends). Statistical tests were performed and P value thresholds were obtained using

GraphPad 5.0.1. or Excel. Comparisons between two groups were performed using two-tailed unpaired Student's t-test. To confirm the reproducibility, every experiment was repeated independently at least three times.

References for Supplementary Methods and Extended Data Figures

Casse, C., Giannoni, F., Nguyen, V.T., Dubois, M.F., and Bensaude, O. (1999). The transcriptional inhibitors, actinomycin D and alpha-amanitin, activate the HIV-1 promoter and favor phosphorylation of the RNA polymerase II C-terminal domain. The Journal of biological chemistry *274*, 16097-16106.

Chu, F.F., Esworthy, R.S., Chu, P.G., Longmate, J.A., Huycke, M.M., Wilczynski, S., and Doroshow, J.H. (2004). Bacteria-induced intestinal cancer in mice with disrupted Gpx1 and Gpx2 genes. Cancer Res *64*, 962-968.

Franco, R., and Cidlowski, J.A. (2009). Apoptosis and glutathione: beyond an antioxidant. Cell death and differentiation *16*, 1303-1314.

Guo, Z., Kozlov, S., Lavin, M.F., Person, M.D., and Paull, T.T. (2010). ATM activation by oxidative stress. Science *330*, 517-521.

Harris, I.S., Treloar, A.E., Inoue, S., Sasaki, M., Gorrini, C., Lee, K.C., Yung, K.Y., Brenner, D., Knobbe-Thomsen, C.B., Cox, M.A., *et al.* (2015). Glutathione and thioredoxin antioxidant pathways synergize to drive cancer initiation and progression. Cancer cell *27*, 211-222.

Kim, K., Doi, A., Wen, B., Ng, K., Zhao, R., Cahan, P., Kim, J., Aryee, M.J., Ji, H., Ehrlich, L.I., *et al.* (2010). Epigenetic memory in induced pluripotent stem cells. Nature *467*, 285-290.

Kim, K., Zhao, R., Doi, A., Ng, K., Unternaehrer, J., Cahan, P., Huo, H., Loh, Y.H., Aryee, M.J., Lensch, M.W., *et al.* (2011). Donor cell type can influence the epigenome and differentiation potential of human induced pluripotent stem cells. Nature biotechnology *29*, 1117-1119.

Koh, E.Y., Chen, T., and Daley, G.Q. (2002). Novel retroviral vectors to facilitate expression screens in mammalian cells. Nucleic Acids Res *30*, e142.

Moran, J.L., Bolton, A.D., Tran, P.V., Brown, A., Dwyer, N.D., Manning, D.K., Bjork, B.C., Li, C., Montgomery, K., Siepka, S.M., *et al.* (2006). Utilization of a whole genome SNP panel for efficient genetic mapping in the mouse. Genome research *16*, 436-440.

Rass, E., Chandramouly, G., Zha, S., Alt, F.W., and Xie, A. (2013). Ataxia telangiectasia mutated (ATM) is dispensable for endonuclease I-SceI-induced homologous recombination in mouse embryonic stem cells. The Journal of biological chemistry *288*, 7086-7095.

Sharova, L.V., Sharov, A.A., Nedorezov, T., Piao, Y., Shaik, N., and Ko, M.S. (2009). Database for mRNA half-life of 19 977 genes obtained by DNA microarray analysis of pluripotent and differentiating mouse embryonic stem cells. DNA research : an international journal for rapid publication of reports on genes and genomes *16*, 45-58.

Skamagki M., C.C., Yeung P., Baslan T., Beck S., Zhang C., Ross C.A., Dang L., Liu Z, Giunta S., Chang T.P., Wang J., Ananthanarayanan A., Bohndorf M., Bosbach B., Adjaye J., Funabiki H., Kim J., Lowe S, Collins J.J., Lu C-W, Li H., Zhao R., Kim K. (2017). ZSCAN10 expression corrects the genomic instability of iPSC from aged donors. Nature Cell Biology *(9)*, 1037-1048.

Sleigh, M.J. (1976). The mechanism of DNA breakage by phleomycin in vitro. Nucleic acids research *3*, 891-901.

Takahashi, K., and Yamanaka, S. (2006). Induction of pluripotent stem cells from mouse embryonic and adult fibroblast cultures by defined factors. Cell *126*, 663-676.

Tian, B., Yang, J., and Brasier, A.R. (2012). Two-step cross-linking for analysis of protein-chromatin interactions. Methods in molecular biology *809*, 105-120.

Yu, H.B., Kunarso, G., Hong, F.H., and Stanton, L.W. (2009). Zfp206, Oct4, and Sox2 are integrated components of a transcriptional regulatory network in embryonic stem cells. The Journal of biological chemistry *284*, 31327-31335.
