# Supplementary material for: RNA Exosome Complex-Mediated Control of Redox Status in Pluripotent Stem Cells
Source: Stem Cell Reports. 2017 Oct 10;9(4):1053–61. doi: 10.1016/j.stemcr.2017.08.024 (PMC5639470; doi:10.1016/j.stemcr.2017.08.024)
Supplement: Document S2. Article plus Supplemental Information [file mmc3.pdf]

Stem Cell Reports
Report

OPEN ACCESS

RNA Exosome Complex-Mediated Control of Redox Status in Pluripotent Stem Cells

Maria Skamagki,[1,2] Cheng Zhang,[3] Christian A. Ross,[3] Aparna Ananthanarayanan,[1,2] Zhong Liu,[4] Quanhua Mu,[5] Uttiya Basu,[6] Jiguang Wang,[5] Rui Zhao,[4] Hu Li,[3,*] and Kitai Kim[1,2,*]

[1]Cancer Biology and Genetics Program, The Center for Cell Engineering, The Center for Stem Cell Biology, Memorial Sloan-Kettering Cancer Center, Sloan-Kettering Institute for Cancer Research

[2]Department of Cell and Developmental Biology, Weill Medical College of Cornell University
New York, NY 10065, USA

[3]Department of Molecular Pharmacology & Experimental Therapeutics, Center for Individualized Medicine, Mayo Clinic College of Medicine, Rochester, MN 55902, USA

[4]Department of Biochemistry and Molecular Genetics, Stem Cell Institute, University of Alabama at Birmingham, Birmingham, AL 35294, USA

[5]Divisions of Life Science, Department of Chemical and Biomedical Engineering, School of Engineering, The Hong Kong University of Science and Technology, Clear Water Bay, Kowloon, Hong Kong

[6]Department of Microbiology and Immunology, College of Physicians and Surgeons, Columbia University, New York, NY 10032, USA

*Correspondence: li.hu@mayo.edu (H.L.), kimk@mskcc.org (K.K.)

http://dx.doi.org/10.1016/j.stemcr.2017.08.024

SUMMARY

The RNA exosome complex targets AU-rich element (ARE)-containing mRNAs in eukaryotic cells. We identified a transcription factor, ZSCAN10, which binds to the promoters of multiple RNA exosome complex subunits in pluripotent stem cells to maintain subunit gene expression. We discovered that induced pluripotent stem cell clones generated from aged tissue donors (A-iPSC) show poor expression of ZSCAN10, leading to poor RNA exosome complex expression, and a subsequent elevation in ARE-containing RNAs, including glutathione peroxidase 2 (*Gpx2*). Excess GPX2 leads to excess glutathione-mediated reactive oxygen species scavenging activity that blunts the DNA damage response and apoptosis. Expression of ZSCAN10 in A-iPSC recovers RNA exosome gene expression, the DNA damage response, and apoptosis. These findings reveal the central role of ZSCAN10 and the RNA exosome complex in maintaining pluripotent stem cell redox status to support a normal DNA damage response.

INTRODUCTION

The RNA exosome complex is a central ring structure of six core proteins and three RNA-binding domain-containing core proteins (Kilchert et al., 2016; Makino et al., 2015) that removes aberrantly accumulated RNA transcripts to prevent events such as altered splicing (Coy et al., 2013), autoimmune response activation (Eckard et al., 2014), and genomic instability caused by RNA-DNA hybridization (Wahba et al., 2013). The RNA exosome complex is highly conserved in eukaryotic cells and functions in both the nucleus and the cytoplasm (Schmid and Jensen, 2008). Recent reports have shown that the RNA exosome complex targets specific RNA transcripts in response to environmental changes during embryo development (Kilchert et al., 2015). In the cytoplasm, the exosome complex interacts with RNA at A + U sequence-rich elements (AREs), causing rapid degradation of target RNA with significant specificity (Chen et al., 2001; Doma and Parker, 2006). Recently, the targeting specificity of the RNA exosome complex was studied in embryonic stem cells (ESCs) by transcriptome analysis (Pefanis et al., 2015). That study showed that the exosome regulates long non-coding RNA transcripts that function as enhancer RNAs to control gene transcription. We found that induced pluripotent stem cells (iPSCs) (Takahashi and Yamanaka, 2006) derived from older donor cells (A-iPSCs; using mouse skin fibroblasts from donors 1.4 years of age) retain an aging-associated epigenetic signature that is not present in iPSCs derived from young donor cells (Y-iPSCs; using mouse skin fibroblasts from E15.5 embryos to 5-day-old neonates) (Skamagki et al., 2017; Kim et al., 2010, 2011). A-iPSCs show transcriptional alterations compared with Y-iPSCs, including poor expression of the pluripotent factor ZSCAN10. ZSCAN10 is a known zinc finger transcription factor specifically expressed in ESCs and an integrated part of the transcriptional regulatory network with SOX2, OCT4, and NANOG (Yu et al., 2009; Zhang et al., 2006). In this report, we found that ZSCAN10 directly binds to the promoters of RNA exosome subunits to stimulate their expression, and that A-iPSCs show poor expression of core RNA exosome subunits due to poor ZSCAN10 expression, which could result in dysfunctional RNA exosome function in A-iPSCs (Figure S1). We utilized A-iPSCs to test the hypothesis that ZSCAN10 regulates gene transcription in PSCs via the RNA exosome complex, specifically focusing on glutathione peroxidase 2 (GPX2) as a downstream target that influences the DNA damage response pathway.

This is an open access article under the CC BY license (http://creativecommons.org/licenses/by/4.0/).

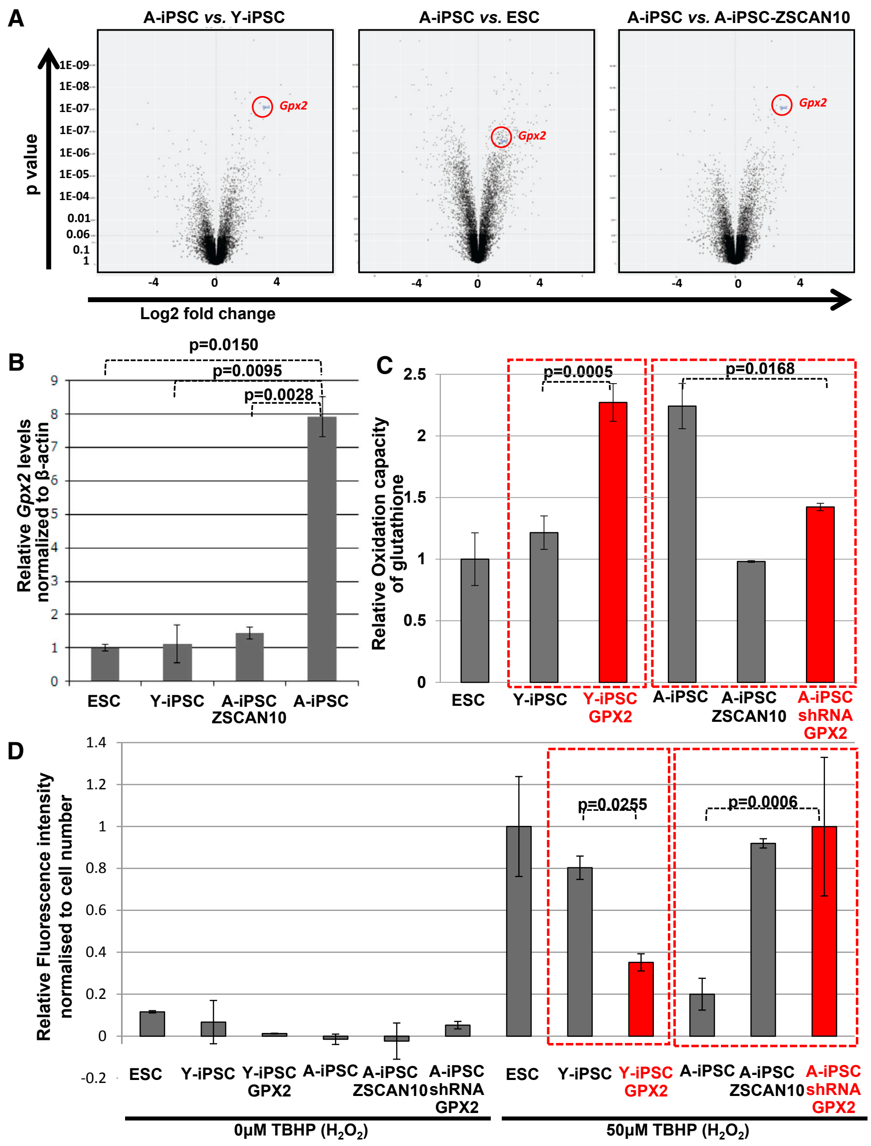

Figure 1. Imbalance of H_20_2/Glutathione Homeostasis in A-iPSCs and Recovery by ZSCAN10 via Reduction of Excessively Activated GPX2

(A) Altered *Gpx2* expression from a comparative gene expression analysis (microarray). *Gpx2* is differentially expressed among the different cell lines (also refer to Figure S4A). At least two clonal repeats (Y-iPSC = 2, A-iPSC = 2, A-iPSC-ZSCAN10 = 4, ESC = 2) were included in the analysis.

(B) qPCR for *Gpx2* indicating increased expression of GPX2 in A-iPSCs and down-regulation by ZSCAN10 expression. Error bars indicate SEM (n = 4).

(C) Quantification of reduced glutathione in ESCs, Y-iPSCs, Y-iPSC-GPX2, A-iPSCs, A-iPSC-ZSCAN10, and A-iPSC-shRNA-GPX2. Increased oxidation capacity of glutathione in A-iPSCs is recovered by reduction of GPX2 via shRNA-GPX2. Conversely, GPX2 overexpression in Y-iPSCs induces elevated glutathione oxidation capacity. Mean ± SD is plotted for four replicates from each condition (n = 4). Statistical significance was determined by two-sided t test.

(D) H_20_2 scavenging activity in ESCs, Y-iPSCs, Y-iPSC-GPX2, A-iPSCs, A-iPSC-ZSCAN10, and A-iPSC-shRNA-GPX2. Increased H_20_2 scavenging activity in A-iPSCs is recovered by shRNA-GPX2 expression. Elevated H_20_2 scavenging activity seen in A-iPSCs can be recapitulated in Y-iPSC by GPX2 overexpression. Mean ± SD is plotted for four replicates from each condition (n = 4). Statistical significance was determined by two-sided t test.

RESULTS

GPX2 Modulates Glutathione Activity and Redox Status in Induced Pluripotent Stem Cells from Young and Aged Donors

We performed a comparative gene expression analysis of ESCs, Y-iPSCs, A-iPSCs, and A-iPSCs expressing ZSCAN10 to identify candidate genes that were up- or downregulated in A-iPSCs compared with A-iPSC-ZSCAN10, and that were expressed at similar levels in A-iPSC-ZSCAN10, ESCs, and Y-iPSCs. We found that the *Gpx2* gene is highly expressed only in A-iPSCs and expression was normalized by ZSCAN10 expression (Figure 1A and S4A), as confirmed by qPCR (Figure 1B). GPX2 supports reactive oxygen species (ROS) scavenging by increasing the reduced form of glutathione (active form) from oxidized glutathione (Chu et al., 2004). We tested the possibility that increased expression of GPX2 in A-iPSCs could induce an imbalance in glutathione-ROS homeostasis. Overexpression of GPX2 in Y-iPSCs (Figure S2A) induced the oxidation capacity of glutathione (Figure 1C). Conversely, short hairpin RNA (shRNA) knockdown of GPX2 in A-iPSCs (Figure S2B) led to a reduction in oxidation capacity of glutathione (Figure 1C). We also observed higher ROS scavenger activity upon GPX2 overexpression in Y-iPSCs (Figure 1D) and reduced ROS scavenger activity with shRNA knockdown of GPX2 in A-iPSCs (Figure 1D).

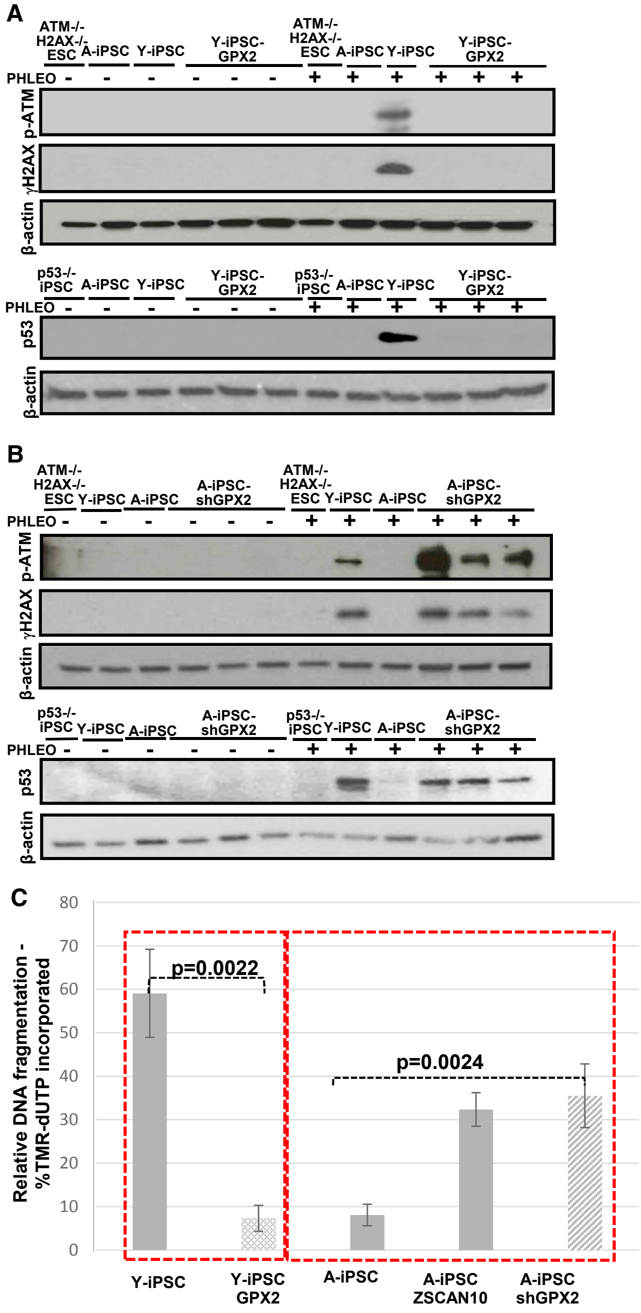

Figure 2. Poor DNA Damage Response and Apoptosis in A-iPSCs and Recovery by ZSCAN10 via Reduction of Excessively Activated GPX2

(A) Immunoblot of pATM/γH2AX/p53 showing an impaired DNA damage response after phleomycin treatment in A-iPSCs and three independent clones of Y-iPSCs with lentiviral expression of GPX2 cDNA (also refer to Figure S2A).

(B) Immunoblot of pATM/γH2AX/p53 showing recovery of the DNA damage response after phleomycin treatment in three independent clones of A-iPSCs with GPX2 shRNA expression (also refer to Figure S2B).

(C) Apoptosis detected by flow cytometry in Y-iPSCs with GPX2 overexpression and A-iPSCs with ZSCAN10 or GPX2 shRNA expression. We observed a lower apoptotic response (DNA fragmentation assay) 15 hr after the end of phleomycin treatment (2 hr, 30 μg/mL) in A-iPSCs and recovery with GPX2 downregulation in A-iPSCs to levels similar to that of A-iPSC-ZSCAN10. Transient expression of GPX2 in Y-iPSCs also reduces apoptotic response to the levels seen in A-iPSCs. Mean ± SD is plotted for multiple replicates (Y-iPSC = 6, Y-iPSC-GPX2 = 3, A-iPSC = 3, A-iPSC-ZSCAN10 = 3, A-iPSC-shGPX2 = 4). Statistical significance was determined by two-sided t test (also refer to Figure S4C) (three independent experiments).

GPX2 Regulates DNA Damage Response and Apoptosis in Pluripotent Stem Cells

These data suggest that the elevated glutathione activity and lower ROS levels in A-iPSCs are related to higher *Gpx2* expression, which leads to a homeostatic imbalance between ROS and glutathione that impairs the DNA damage response (Skamagki et al., 2017). Therefore, we investigated whether GPX2 overexpression is one of the main drivers of the increased glutathione and the blunted DNA damage response in A-iPSCs. We found that shRNA knockdown of GPX2 in A-iPSCs recovered the DNA damage response, as indicated by activation of the pATM, γH2AX, and p53 pathways (Figure 2B). Conversely, overexpression of GPX2 in Y-iPSCs blunted the DNA damage response (Figure 2A). GPX2 overexpression and lower ROS in Y-iPSCs was correlated with significantly reduced apoptosis (Figures 2C and S4C) (Franco and Cidlowski, 2009) and poor activation of the DNA damage response pathway (Guo et al., 2010; Sleigh, 1976). shRNA knockdown of GPX2 and higher ROS in A-iPSCs recovered the apoptosis rate equivalent to that of Y-iPSCs (Figures 2C and S4C) with reactivation of the DNA damage response pathway.

ZSCAN10 Targets Exosome Subunits to Maintain ARE-Mediated *GPX2* RNA Reduction

From a comparative gene expression analysis of ESCs, Y-iPSCs, A-iPSCs, and A-iPSCs expressing ZSCAN10, we observed that ZSCAN10 expression normalized *Gpx2* expression in A-iPSCs, and high GPX2 expression was associated with higher ROS scavenging activity and a blunted DNA damage response. That led us to hypothesize that the PSC-specific transcription factor ZSCAN10 directly binds to the *Gpx2* promoter (Yu et al., 2009), but could not find any binding activity near the *Gpx2* genomic region. However, we found that *Gpx2* contains four AREs, which are targeted by ARE-binding proteins to the RNA exosome complex. AREs were defined by measuring the decay rate of the RNA with the specific target consensus DNA sequence (Zubiaga et al., 1995).

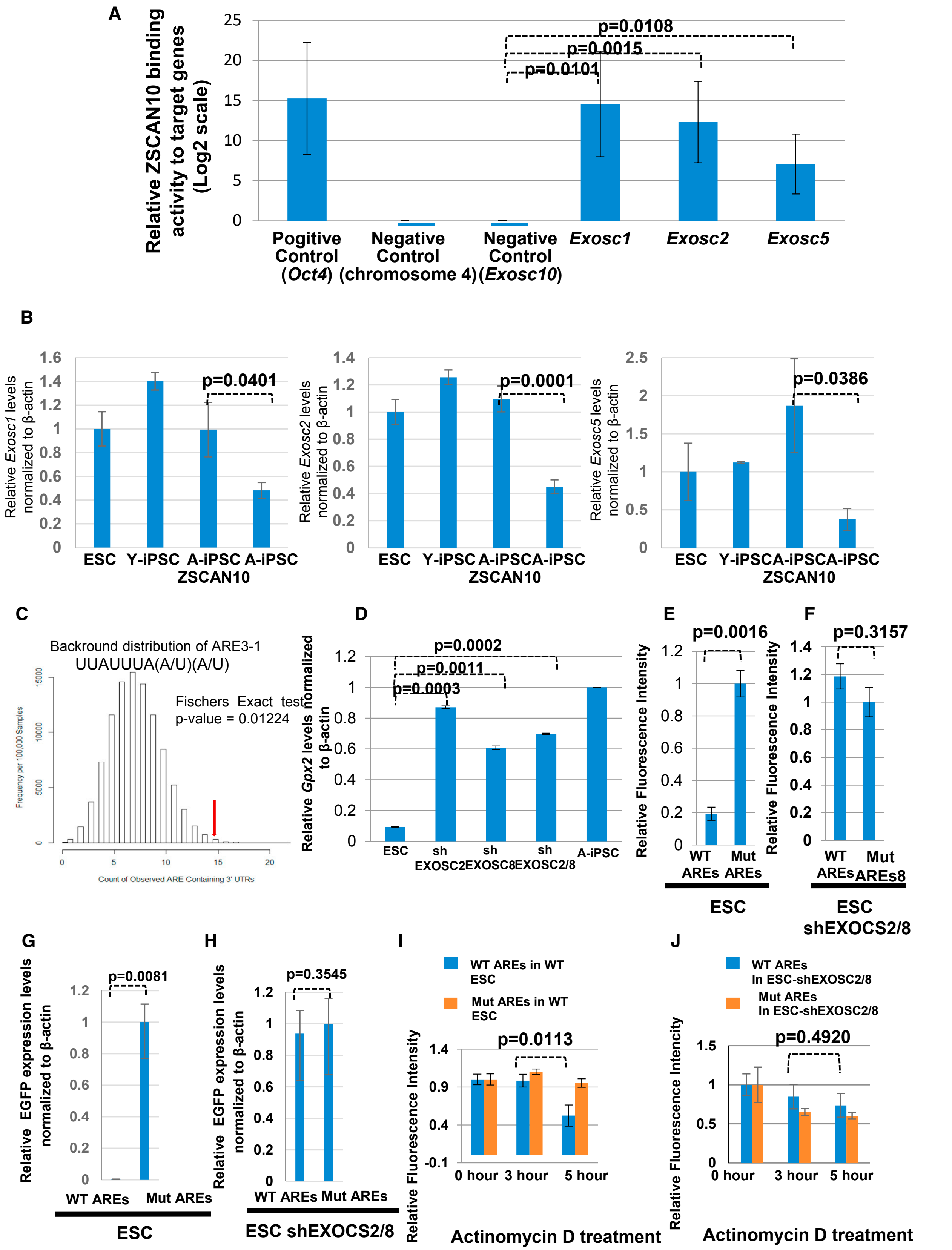

(legend on next page)

Therefore, we hypothesized that ZSCAN10 regulates *Gpx2* expression indirectly by controlling RNA exosome complex subunit gene expression. Chromatin immunoprecipitation (ChIP)-on-Chip data showed strong binding activity of ZSCAN10 to the promoters of multiple exosome complex subunits, including *Exosc1/2/5* (Yu et al., 2009). We generated a biotin-tagged ZSCAN10-tomato fluorescent protein reporter in ESCs and performed ZSCAN10 ChIP-qPCR analysis. We found that ZSCAN10 binds to multiple EXOSC subunits, including EXOSC1/2/5, but had no binding activity to *Exosc10* as a negative control, thus confirming the published ChIP-on-Chip data (Yu et al., 2009) (Figure 3A). qPCR analysis of *Exosc*1/2/5 expression in ESCs/Y-iPSCs/A-iPSCs/A-iPSC-ZSCAN10 showed that relatively higher levels of *Zscan10* expression in ESCs/Y-iPSCs were correlated with higher levels of *Exosc1/2/5* gene expression (Figures 3B and S4B), whereas the lower levels of ZSCAN10 in A-iPSCs were correlated with lower levels of *Exosc*1/2/5 gene expression (Figures 3B and S4B). Furthermore, overexpression of ZCAN10 in A-iPSCs led to restoration of *Exosc1/2/5* gene expression (Figures 3B and S4B), demonstrating a mechanistic link between ZSCAN10 and exosome subunit expression. ARE consensus sequences are well studied, and functional inhibitory ARE consensus sequences based on comparative RNA stability analysis have been identified (Zubiaga et al., 1995). We hypothesized that if A-iPSCs contain fewer core RNA exosome subunits (and thus fewer functional RNA exosome complexes), we would expect to see a slower turnover of ARE-containing transcripts. To test this hypothesis, we performed gene enrichment analysis of a gene set containing the minimal AU-rich motif targeted by the RNA exosome complex that effectively destabilizes mRNA (Sharova et al., 2009; Zubiaga et al., 1995) among a list of genes highly expressed in A-iPSCs (poor expression of ZSCAN10) and in ESCs, Y-iPSCs, and A-iPSC-ZSCAN10 (normal ZSCAN10 expression level; Figure 3C). We observed significant ARE-containing gene enrichment in A-iPSCs ($p = 0.012$) (Figure 3C), suggesting that loss of ZSCAN10-mediated RNA exosome complex subunit expression in A-iPSCs allows significant upregulation of ARE-containing RNAs (Table S1). *Gpx2* was one of the functional ARE-containing transcripts upregulated in A-iPSCs. As a control, we also performed the gene enrichment analysis with a subset of ARE-containing sequences that did not show functional effects in the previous report

Figure 3. ZSCAN10 Targets the RNA Exosome Complex, which Influences *GPX2* Gene Expression

(A) Confirmation of ZSCAN10 binding activity to the exosome subunits reported by Yu et al. (2009) using ChIP-qPCR. The graph shows the fold enrichment compared with a negative control and normalized by input. Error bars indicate the SEM of three replicates (n = 3). Statistical significance was determined by two-sided t test. The *Oct4* promoter region was used as a positive control and an 80 bp region on chromosome 4 was used as a negative control. The *Exosc10* region that did not show ZSCAN10 binding activity was used as an additional negative control.

(B) Correlation between *Zscan10* expression levels and RNA exosome subunit expression. Error bars indicate SEM. Multiple independent replicates in each samples (A-iPSC = 5, A-iPSC-ZSCAN10 = 6, Y-iPSC = 3, ESC = 3). Statistical significance was determined by two-sided t test (also refer to Figure S4B).

(C) Enrichment analysis for ARE3-1[UUAUUUA[AU][AU]] in the 3′ UTR (Zubiaga et al., 1995) of a set of genes/transcripts upregulated in A-iPSCs compared with ESCs/Y-iPSCs/A-iPSC-ZSCAN10. The comparison was performed against a control whole-genome transcript pool (Sharova et al., 2009). The vertical red line indicates overlap of 14 such transcripts out of 60 interrogated. The histogram represents a random probability distribution of overlap ($p = 0.012$; 100,000 permutations). Statistical significance was estimated by Fisher's exact test.

(D) Gene expression analysis of *Gpx2* mRNA with shRNA knockdown of EXOSC2 and/or EXOSC8 by qPCR. The *Gpx2* mRNA level is low in ESCs, and shRNA knockdown of EXOSC2 and/or EXOSC8 significantly increases *Gpx2* expression. Mean ± SD is plotted for three replicates in each sample group from each condition (n = 3). Statistical significance was determined by two-sided t test.

(E) Low expression in ESCs of a destabilized form of GFP reporter construct containing wild-type GPX2 AREs and recovery of expression after introduction of mutations in the GPX2 AREs (n = 3).

(F) Recovery of wild-type GPX2 ARE reporter construct in ESCs expressing shEXOSC2/8. Mean ± SD is plotted for three replicates in each sample group from each condition (three independent experiments). Statistical significance was determined by two-sided t test (n = 3).

(G and H) The expression level of the destabilized EGFP reporter was further confirmed using qPCR to measure the mRNA levels of EGFP under the control of wild-type (WT) and mutant (MUT) GPX2 ARE sequences in both ESCs (G) and ESCs expressing shRNA EXOSC2/8 (H). The RNA samples were collected 5 hr post actinomycin D treatment (0.3 μg/mL). Fold changes in EGFP under the control of WT-GPX2-ARE are shown relative to EGFP mRNA levels under the control of MUT-GPX2-ARE. Fold change is plotted for three replicates for each group from each condition (n = 3). Statistical significance was determined by two-sided t test. Error bars indicate SEM.

(I) Measurement of GFP reporter expression rate for a reporter containing wild-type GPX2 AREs versus mutant GPX2 AREs. RNA degradation rate was monitored with inhibition of transcription by actinomycin D treatment.

(J) Recovery of rapid reduction in expression of the wild-type GPX2 ARE GFP reporter in ESCs expressing shEXOSC2/8. Mean ± SD is plotted for three replicates in each sample group from each condition (n = 3) (three independent experiments). Statistical significance was determined by two-sided t test.

(Zubiaga et al., 1995), and confirmed no significant enrichment (Figure S2C).

Modulation of the RNA Exosome Complex Influences *GPX2* Gene Expression

Among the gene list (Table S1), the *Gpx2* gene contains multiple ARE sequences, suggesting a mechanism by which ZSCAN10, via the RNA exosome complex, could regulate GPX2 and the redox status of iPSCs. To test this hypothesis, we could modify expression levels of EXOSC1/2/5 in A-iPSCs equivalent to the gene expression level of ESCs/Y-iPSCs. Thus, we knocked down the expression of individual RNA exosome complex subunits in ESCs and monitored the levels of *Gpx2* gene expression. Because the exosome complex requires assembly of multiple subunits, the knockdown of a single core complex subunit can affect its function (Boczonadi et al., 2014; Flynn et al., 2011; Pefanis et al., 2014). We chose to knock down EXOSC2 because it is the subunit that is downregulated to the greatest extent in A-iPSCs, and it is required for stabilization of the hexameric ring of RNase PH-domain subunits by interacting with EXOSC4 and EXOSC7 (van Dijk et al., 2007). We disrupted the exosome complex by shRNA knockdown of EXOSC2 in ESCs (Figure S3), which led to a dramatic increase in *Gpx2* gene expression (Figure 3D), similar to the expression level in A-iPSCs (Figure 3D). To monitor independent disruption of the exosome complex function by knockdown of an unrelated exosome subunit, we also knocked down EXOSC8 expression (Figure S3), which is known to upregulate ARE-containing RNAs (Boczonadi et al., 2014), but is not targeted by ZSCAN10 in PSCs. We observed that shRNA knockdown of EXOSC8 also enhances *Gpx2* gene expression (Figure 3D). In addition, shRNA knockdown of both EXOSC2 and EXOSC8 elevated *Gpx2* expression but did not show an additive effect (Figure 3D), confirming that the knockdown of a single exosome subunit is sufficient to disrupt RNA exosome complex function. The mechanistic link between the RNA exosome complex and *Gpx2* expression was further confirmed in ESCs by the poor expression of a GFP (a destabilized form of GFP) reporter construct containing wild-type GPX2 AREs and recovery of reporter expression after introduction of mutations in the GPX2 AREs (Figure 3E). Expression of the wild-type GPX2 ARE reporter in ESCs was also recovered with expression of EXOSC2/8 shRNA (Figure 3F). Inhibition of transcription by actinomycin D treatment led to a faster reduction in wild-type GPX2 ARE reporter expression than the mutant GPX2 ARE reporter expression (Figure 3I). Again, wild-type GPX2 ARE reporter expression could be rescued in ESCs expressing EXOSC2/8 shRNA (Figure 3J). The expression of wild-type and mutant GPX2 ARE GFP reporters in ESCs, with or without EXOSC2/8 shRNA, was confirmed by qPCR (Figures 3G and 3H).

RNA Exosome Complex Controls GPX2 to Maintain Redox Homeostasis and DNA Damage Response

Because *Gpx2* gene expression was increased by knockdown of RNA exosome complex subunit gene expression, we hypothesized that knockdown of exosome subunits by shRNA would also reduce the apoptotic response to exogenous stress (phleomycin treatment) in ESCs, similar to the effect of GPX2 overexpression in Y-iPSCs. Indeed, knockdown of EXOSC2 or EXOSC8 in ESCs significantly reduced the apoptotic response to stress (Figures 4A and 4B), with no significant additive effect of knocking down both EXOSC2 and EXOSC8 (Figures 4A and 4B). We further assessed the effect of EXOSC2 knockdown on the homeostatic imbalance between glutathione and ROS and the DNA damage response in ESCs. Similar to what was seen with overexpression of GPX2 in Y-iPSCs, knockdown of EXOSC2 in ESCs elevated the oxidation capacity of glutathione and reduced ROS scavenging activity (Figures 4C, 4D, and S4D). In addition, the DNA damage response was disrupted by EXOSC2 knockdown (Figure 4E), indicating that the RNA exosome complex has a significant role in regulating *Gpx2* expression levels to maintain the balance between glutathione and ROS and support a normal DNA damage response.

DISCUSSION

In summary, we found that a pluripotent transcription factor, ZSCAN10, binds near the transcription start sites of multiple RNA exosome complex subunits (*Exosc*1/2/5) in PSCs, as reported previously (Yu et al., 2009). Poor expression of ZSCAN10 in A-iPSCs led to poor exosome subunit expression and an increase in ARE-containing RNAs, including *Gpx2*. Elevated GPX2 in turn increases the reduced form of glutathione (active form), which scavenges ROS, blunts the DNA damage response, and reduces apoptosis. Expression of ZSCAN10 in A-iPSCs normalizes RNA exosome subunit expression, which reduces GPX2 and restores glutathione homeostasis and the DNA damage response. Taken together, these results provide mechanistic insights into the role of the RNA exosome regulatory pathway in PSCs, as well as the regulation of the RNA exosome complex itself by the pluripotent factor ZSCAN10 (Figure S1). Here, we report that the PSC-specific transcription factor ZSCAN10 can sustain the expression level of the RNA exosome complex subunits 1/2/5 in PSCs, and that knockdown of EXOSC2 and/or EXOSC8 causes similar effects in ESCs, with no significant additive effect of knocking down the combination. This suggests that each subunit is necessary for the function of the entire RNA exosome complex. However, certain exosome subunit mutations have been found to be more dominant and cause specific

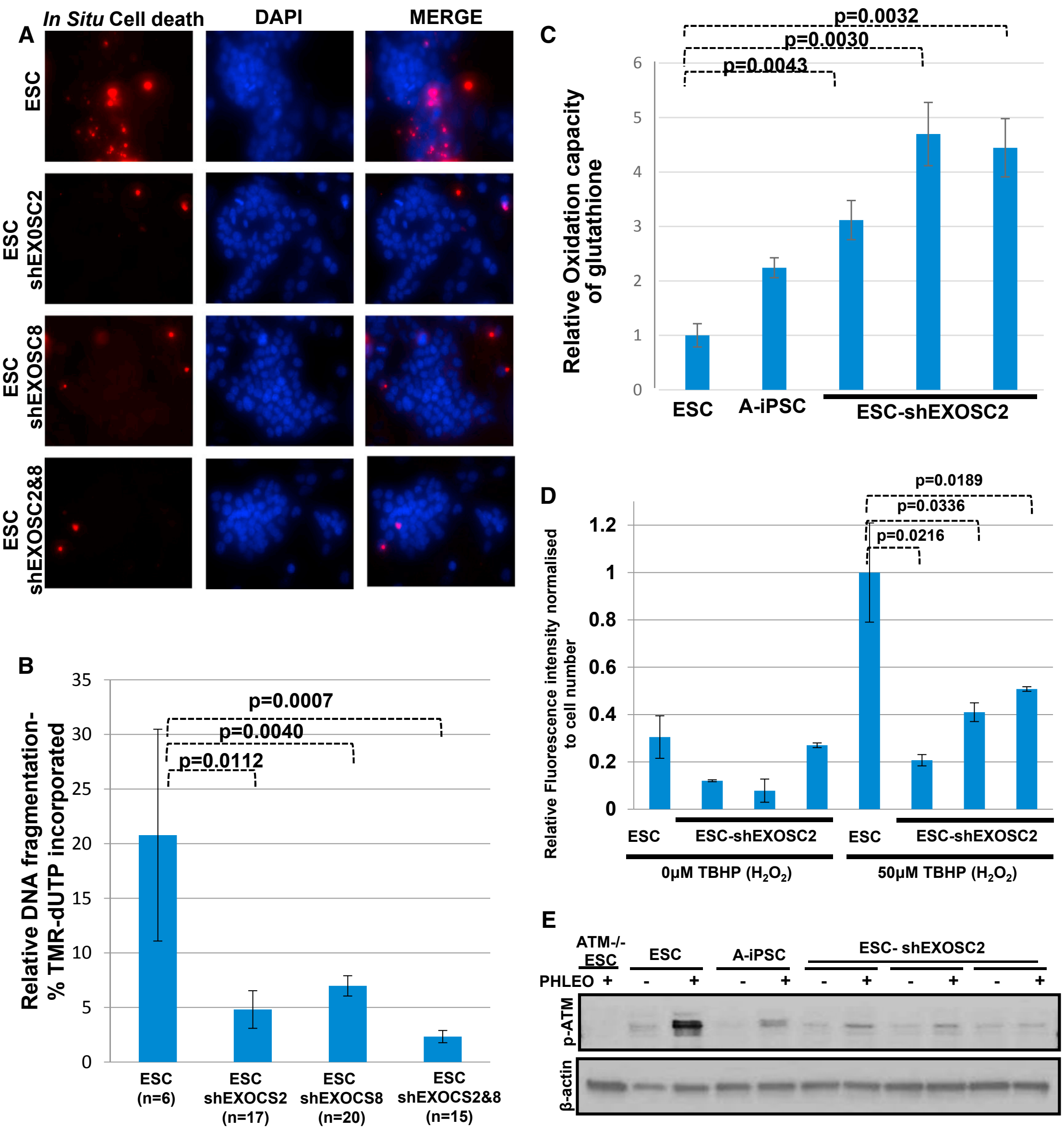

Figure 4. Recapitulation of poor Apoptosis, Imbalance of ROS/Glutathione Homeostasis, and Poor DNA Damage Response in ESCs by shRNA EXOSC2/8 Expression

(A) *In situ* cell death assays of ESCs with or without shRNA for EXOSC2, EXOSC8, or both EXOSC2/EXOSC8 were performed 15 hr after the end of phleomycin treatment (2 hr, 30 μg/mL). ESCs with either EXOSC2 shRNA, EXOSC8 shRNA, or both shRNAs show fewer cells staining for cell death compared with ESCs. Nuclei are stained with DAPI. Scale bar indicates 100 μm.

(B) Quantification by image analysis of the apoptotic response in the DNA fragmentation assay after phleomycin treatment. Error bars indicate the SEM of multiple replicates (ESC = 8, ESC-shEXOSC2 = 17, ESC-shEXOSC8 = 20, ESC-shEXOSC2 and 8 = 15). Statistical significance was determined by unpaired two-sided t test.

(C) Quantification of the reduced form of glutathione in ESCs and ESCs with EXOSC2 shRNA. Mean ± SD is plotted for multiple replicates from each condition (ESC = 4, A-iPSC = 6, ESC-shEXOSC2 cl.1 = 3, ESC-shEXOSC2 cl.2 = 3, ESC-shEXOSC2 cl.3 = 3) (three independent experiments). Statistical significance was determined by two-sided t test (also refer to Figure S4D).

(D) H_2O_2 scavenging activity in ESCs and ESCs with either EXOSC2, EXOSC8, or EXOSC2/EXOSC8 shRNA. Mean ± SD is plotted for three replicates in each sample group from each condition (n = 3) (three independent experiments). Statistical significance was determined by two-sided t test.

(E) Immunoblot of pATM/γH2AX/p53 shows impaired DNA damage response after phleomycin treatment in ESCs and ESCs with either EXOSC2, EXOSC8, or EXOSC2/EXOSC8 shRNA.

phenotypes. For example, mutations in *Exosc2* are specifically associated with a syndrome characterized by retinitis pigmentosa, progressive hearing loss, premature aging, short stature, mild intellectual disability, and distinctive gestalt (Di Donato et al., 2016). This may indicate that *Exosc2* mutations have a more deleterious effect than mutations in other exosome subunits. Another possibility is that each exosome subunit mutation causes a selective advantage to target specific ARE-containing RNAs through alterations in the overall exosome complex. Additional biological roles of the specific RNA exosome complex subunits *Exosc*1/2/5, and their regulation by ZSCAN10 in PSCs, remain to be studied. With regard to ZSCAN10 as a regulator of the exosome complex, it would be interesting to identify the upstream factors that regulate the differential activation of ZSCAN10 in Y-iPSCs and A-iPSCs. The mechanism may involve differential epigenetic regulation, given that the cell lines used here have the same genetic background. While epigenetic regulation can be altered by aging, it may be challenging to directly connect differences in ZSCAN10 activation to differences in somatic cell epigenetic memory, because ZSCAN10 is only expressed in PSCs. As increasing the passage number did not recover ZSCAN10 expression in A-iPSCs, we are currently investigating epigenetic mechanisms upstream of ZSCAN10 that may regulate its expression. An indirect influence of the accumulation of epigenetic modifications with aging might lead to poor activation of ZSCAN10 in A-iPSCs versus Y-iPSCs. Once the mechanism of ZSCAN10 activation has been deciphered, the next step would be to identify small molecules that can directly affect these pathways and explore their potential for improving A-iPSC quality. ZSCAN10-targeted genes have been reported by Yu et al. (2009) using ChIP-on-Chip analysis. Genes that are directly or indirectly regulated by ZSCAN10 need to be validated and their specific functional roles identified.

EXPERIMENTAL PROCEDURES

ESCs and iPSCs were cultured in ESC medium containing 20% fetal bovine serum and 1,000 U/mL of leukemia inhibitory factor. For reprogramming of somatic cells, retroviruses expressing OCT4, SOX2, KLF4, and MYC were introduced. Information regarding cell lines, antibodies, plasmids, and drugs used in this study, as well as detailed protocols for reprogramming and cell culture, overexpression and knockdown, gene expression analysis (qPCR), immunofluorescence staining and analysis, immunoblot analysis, retrovirus and lentivirus production, drug treatments, glutathione detection assay, and H_2O_2 ROS assay are provided in the Supplemental Experimental Procedures. The animal study is compliant with all relevant ethical regulations regarding animal research by an Institutional Animal Care and Use Committee (IACUC approval number 11-10-023).

ACCESSION NUMBERS

Microarray data that support the findings of this study have been deposited in the Gene Expression Omnibus under accession code GEO: GSE85365.

SUPPLEMENTAL INFORMATION

Supplemental Information includes Supplemental Experimental Procedures, four figures, and one table and can be found with this article online at http://dx.doi.org/10.1016/j.stemcr.2017.08.024.

AUTHOR CONTRIBUTIONS

M.S., R.Z., and K.K. conceived the experimental plan. M.S., A.A., and Z.L. performed the experiments. C.Z., C.A.R., Q.M., J.W., and H.L. performed the computational analysis. M.S., C.Z., C.A.R., Z.L., Q.M., U.B., J.W., R.Z., H.L., and K.K. wrote the manuscript.

ACKNOWLEDGMENTS

K.K. is supported by NIH (R00HL093212 and R01AG043531), TriStem-Star Foundation (2013-049), Louis V. Gerstner, Jr. Young Investigators awards, Geoffrey Beene Junior Chair Award, Sidney Kimmel Scholar Award, Alfred W. Bressler Scholars Endowment Fund, and MSKCC Society Fund. MSKCC Molecular Cytogenetics Core was supported by an NIH Cancer Center support grant P30 CA008748. H.L. is supported by the Mayo Clinic Center for Individualized Medicine, the Mayo Clinic Center for Regenerative Medicine, and by a grant from the Paul F. Glenn Foundation. R.Z. is supported by the UAB Development Fund. U.B. is supported by NIAID (2R01AI099195-06). J.W. is supported by HKUST initiation grants.

Received: May 19, 2017
Revised: August 29, 2017
Accepted: August 30, 2017
Published: October 10, 2017

REFERENCES

Boczonadi, V., Muller, J.S., Pyle, A., Munkley, J., Dor, T., Quartararo, J., Ferrero, I., Karcagi, V., Giunta, M., Polvikoski, T., et al. (2014). EXOSC8 mutations alter mRNA metabolism and cause hypomyelination with spinal muscular atrophy and cerebellar hypoplasia. Nat. Commun. *5*, 4287.

Chen, C.Y., Gherzi, R., Ong, S.E., Chan, E.L., Raijmakers, R., Pruijn, G.J., Stoecklin, G., Moroni, C., Mann, M., and Karin, M. (2001). AU binding proteins recruit the exosome to degrade ARE-containing mRNAs. Cell *107*, 451–464.

Chu, F.F., Esworthy, R.S., Chu, P.G., Longmate, J.A., Huycke, M.M., Wilczynski, S., and Doroshow, J.H. (2004). Bacteria-induced intestinal cancer in mice with disrupted Gpx1 and Gpx2 genes. Cancer Res. *64*, 962–968.

Coy, S., Volanakis, A., Shah, S., and Vasiljeva, L. (2013). The Sm complex is required for the processing of non-coding RNAs by the exosome. PLoS One *8*, e65606.

Di Donato, N., Neuhann, T., Kahlert, A.K., Klink, B., Hackmann, K., Neuhann, I., Novotna, B., Schallner, J., Krause, C., Glass, I.A., et al. (2016). Mutations in EXOSC2 are associated with a novel syndrome characterised by retinitis pigmentosa, progressive hearing loss, premature ageing, short stature, mild intellectual disability and distinctive gestalt. J. Med. Genet. *53*, 419–425.

Doma, M.K., and Parker, R. (2006). Endonucleolytic cleavage of eukaryotic mRNAs with stalls in translation elongation. Nature *440*, 561–564.

Eckard, S.C., Rice, G.I., Fabre, A., Badens, C., Gray, E.E., Hartley, J.L., Crow, Y.J., and Stetson, D.B. (2014). The SKIV2L RNA exosome limits activation of the RIG-I-like receptors. Nat. Immunol. *15*, 839–845.

Flynn, R.A., Almada, A.E., Zamudio, J.R., and Sharp, P.A. (2011). Antisense RNA polymerase II divergent transcripts are P-TEFb dependent and substrates for the RNA exosome. Proc. Natl. Acad. Sci. USA *108*, 10460–10465.

Franco, R., and Cidlowski, J.A. (2009). Apoptosis and glutathione: beyond an antioxidant. Cell Death Differ. *16*, 1303–1314.

Guo, Z., Kozlov, S., Lavin, M.F., Person, M.D., and Paull, T.T. (2010). ATM activation by oxidative stress. Science *330*, 517–521.

Kilchert, C., Wittmann, S., Passoni, M., Shah, S., Granneman, S., and Vasiljeva, L. (2015). Regulation of mRNA levels by decay-promoting introns that recruit the exosome specificity factor Mmi1. Cell Rep. *13*, 2504–2515.

Kilchert, C., Wittmann, S., and Vasiljeva, L. (2016). The regulation and functions of the nuclear RNA exosome complex. Nat. Rev. *17*, 227–239.

Kim, K., Doi, A., Wen, B., Ng, K., Zhao, R., Cahan, P., Kim, J., Aryee, M.J., Ji, H., Ehrlich, L.I., et al. (2010). Epigenetic memory in induced pluripotent stem cells. Nature *467*, 285–290.

Kim, K., Zhao, R., Doi, A., Ng, K., Unternaehrer, J., Cahan, P., Huo, H., Loh, Y.H., Aryee, M.J., Lensch, M.W., et al. (2011). Donor cell type can influence the epigenome and differentiation potential of human induced pluripotent stem cells. Nat. Biotechnol. *29*, 1117–1119.

Makino, D.L., Schuch, B., Stegmann, E., Baumgartner, M., Basquin, C., and Conti, E. (2015). RNA degradation paths in a 12-subunit nuclear exosome complex. Nature *524*, 54–58.

Pefanis, E., Wang, J., Rothschild, G., Lim, J., Chao, J., Rabadan, R., Economides, A.N., and Basu, U. (2014). Noncoding RNA transcription targets AID to divergently transcribed loci in B cells. Nature *514*, 389–393.

Pefanis, E., Wang, J., Rothschild, G., Lim, J., Kazadi, D., Sun, J., Federation, A., Chao, J., Elliott, O., Liu, Z.P., et al. (2015). RNA exosome-regulated long non-coding RNA transcription controls super-enhancer activity. Cell *161*, 774–789.

Schmid, M., and Jensen, T.H. (2008). The exosome: a multipurpose RNA-decay machine. Trends Biochem. Sci. *33*, 501–510.

Sharova, L.V., Sharov, A.A., Nedorezov, T., Piao, Y., Shaik, N., and Ko, M.S. (2009). Database for mRNA half-life of 19 977 genes obtained by DNA microarray analysis of pluripotent and differentiating mouse embryonic stem cells. DNA Res. *16*, 45–58.

Skamagki, M., Correia, C., Yeung, P., Baslan, T., Beck, S., Zhang, C., Ross, C.A., Dang, L., Liu, Z., Giunta, S., et al. (2017). ZSCAN10 expression corrects the genomic instability of iPSC from aged donors. Nat. Cell Biol. *19*, 1037–1048.

Sleigh, M.J. (1976). The mechanism of DNA breakage by phleomycin in vitro. Nucleic Acids Res. *3*, 891–901.

Takahashi, K., and Yamanaka, S. (2006). Induction of pluripotent stem cells from mouse embryonic and adult fibroblast cultures by defined factors. Cell *126*, 663–676.

van Dijk, E.L., Schilders, G., and Pruijn, G.J. (2007). Human cell growth requires a functional cytoplasmic exosome, which is involved in various mRNA decay pathways. RNA *13*, 1027–1035.

Wahba, L., Gore, S.K., and Koshland, D. (2013). The homologous recombination machinery modulates the formation of RNA-DNA hybrids and associated chromosome instability. Elife *2*, e00505.

Yu, H.B., Kunarso, G., Hong, F.H., and Stanton, L.W. (2009). Zfp206, Oct4, and Sox2 are integrated components of a transcriptional regulatory network in embryonic stem cells. J. Biol. Chem. *284*, 31327–31335.

Zhang, W., Walker, E., Tamplin, O.J., Rossant, J., Stanford, W.L., and Hughes, T.R. (2006). Zfp206 regulates ES cell gene expression and differentiation. Nucleic Acids Res. *34*, 4780–4790.

Zubiaga, A.M., Belasco, J.G., and Greenberg, M.E. (1995). The nonamer UUAUUUAUU is the key AU-rich sequence motif that mediates mRNA degradation. Mol. Cell Biol. *15*, 2219–2230.

Stem Cell Reports, Volume *9*

Supplemental Information

RNA Exosome Complex-Mediated Control of Redox Status in Pluripotent Stem Cells

Maria Skamagki, Cheng Zhang, Christian A. Ross, Aparna Ananthanarayanan, Zhong Liu, Quanhua Mu, Uttiya Basu, Jiguang Wang, Rui Zhao, Hu Li, and Kitai Kim

Figure. S1. Schematic regulatory model of the homeostatic balance between ROS and glutathione by the RNA exosome complex. (Related to Figures 1-4) We previously found that A-iPSC show transcriptional alterations, including poor expression of the pluripotent factor ZSCAN10, compared to Y-iPSC (Skamagki et al. 2017). In this study, we report that the pluripotent factor ZSCAN10 binds directly to the promoters of multiple components of the RNA exosome complex and stimulates their expression. Concurrently, we found that the glutathione peroxidase 2 (GPX2) RNA transcript contains AREs targeted by the RNA exosome complex. GPX2 increases the reduced form of glutathione (GSH: active form) from oxidized glutathione (Chu et al. 2004). Glutathione is a scavenger metabolite for reactive oxygen species (ROS), and the homeostatic balance between glutathione and ROS is important to maintain the DNA damage response signalling pathway and maintain genomic stability (Sleigh 1976; Franco and Cidlowski 2009). Loss of the homeostatic balance with lower glutathione causes an excess of ROS, which directly damages DNA. Conversely, because ROS is an important cellular signal of stress that induces the DNA damage response, loss of the homeostatic balance with excessive glutathione depletes ROS and leads to a defective DNA damage response. This increases cell exposure to additional genotoxic stresses, and leads to accumulation of mutations (Sleigh 1976; Guo et al. 2010; Harris et al. 2015). We utilized A-iPSC to test the hypothesis that ZSCAN10 indirectly regulates GPX2 expression in pluripotent stem cells *via* interaction with the RNA exosome complex to maintain the ROS-glutathione balance in pluripotent stem cells. In our proposed model, poor expression of ZSCAN10 in A-iPSC reduces RNA exosome complex subunit expression and consequently limits ARE-mediated RNA degradation, including that of GPX2 transcripts. Consequently, the elevated levels of GPX2 in A-iPSC increase the active form of glutathione, causing a homeostatic imbalance between ROS and glutathione.

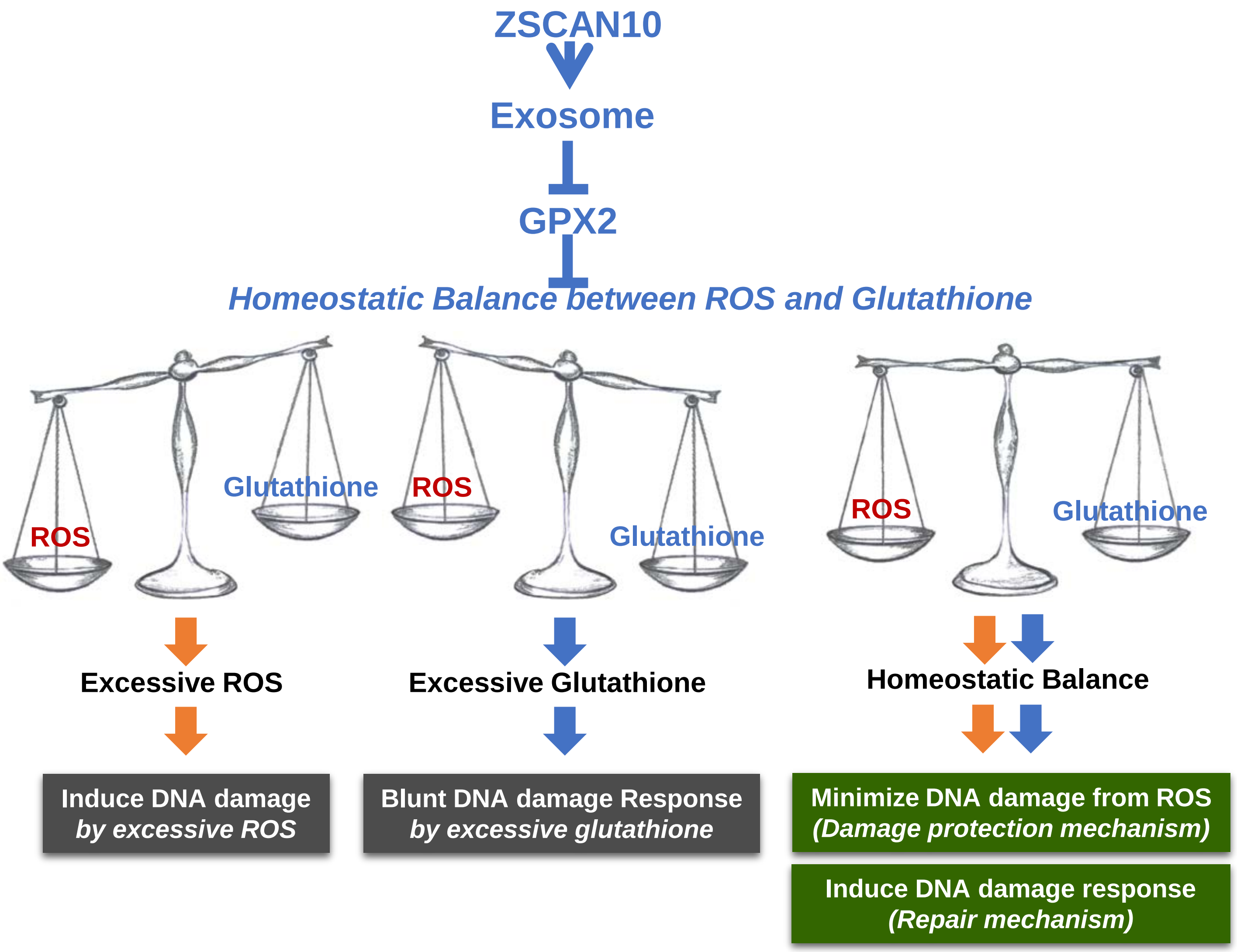

Table S1. Gene enrichment analysis of a gene set containing the minimal AU-rich motif targeted by the RNA exosome complex that effectively destabilizes mRNA transcripts. Upregulated genes in A-iPSC compared to A-iPSC-ZSCAN10 (more than 3-fold), with ARE information (Related **to Figure 3C**; also refer to the independent Excel files). We performed gene enrichment analysis of a gene set containing the minimal AU-rich motif targeted by the RNA exosome complex that effectively destabilizes mRNA transcripts. The gene set included genes that are highly expressed in A-iPSC (poor expression of ZSCAN10) compared to ESC, Y-iPSC, and A-iPSC-ZSCAN10 (normal ZSCAN10 expression level; **Figure 3C**). The comparison was performed against a control whole genome transcript pool (Sharova et al. 2009). We observed significant ARE-containing gene enrichment in A-iPSC (p=0.012) (**Figure 3C**), suggesting that loss of ZSCAN10-mediated RNA exosome complex subunit expression in A-iPSC allows significant upregulation of ARE-containing RNAs (**Table S1**).

Related to Figure 3C Refer the independent Excel files.

Figure S2. Expression level of *Gpx2* after shRNA and cDNA expression and gene enrichment analysis. (Related to Figures 1, 2 & 3C) (A) *Gpx2* expression levels in Y-iPSC after overexpression of GPX2. (B) Expression levels of *Gpx2* after shRNA knockdown in A-iPSC. Mean ± standard deviation is plotted for three independent replicates for *Gpx2* overexpression and shRNA knockdown in each sample group from each condition (n=3). Statistical significance was determined by two-sided t-test. (C) Gene enrichment analysis was performed with ARE1[AUUUA] and/or ARE2-1[UUAUUUAUU] in a set of genes/transcripts that were upregulated in A-iPSC compared to ESC/Y-iPSC/A-iPSC-ZSCAN10. The comparison was performed against a control whole genome transcript pool (Sharova et al. 2009). Vertical red line indicates overlap of 41 such transcripts out of 60 interrogated. The histogram represents a random probability distribution of overlap (100,000 permutations). The figure demonstrates a mathematical point about sampling distributions: that the likelihood of any given transcript to have an ARE is x, so the odds of us seeing this many in a sample based on just random chance is Y. Based on the background of ARE1-2s in the population (13,611/19,977 transcripts), we observed more than half of transcripts have an ARE (41 out of 60 transcripts); therefore, there is no significant enrichment.

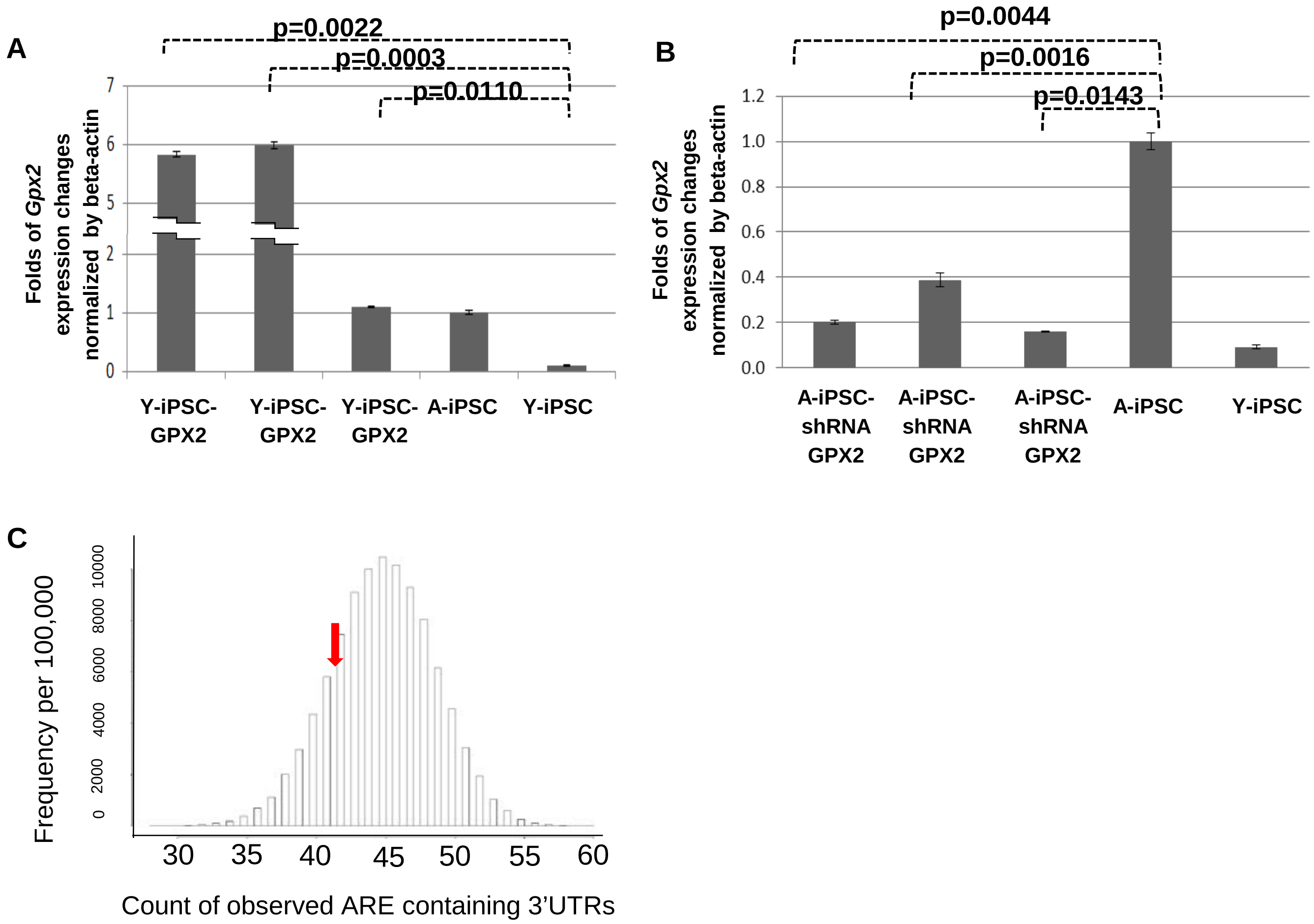

Figure S3. Expression level of *Exosc2* and *Exosc8* after shRNA knockdown in ESC. (Related to Figures 3 and 4) (A) Expression levels of *Exosc2* after shRNA knockdown of EXOSC2 or EXOSC2/8 in ESC. (B) Expression levels of *Exosc8* after shRNA knockdown of EXOSC8 or EXOSC2/8 in ESC. Mean ± standard deviation is plotted for three replicates for ESC shEXOSC2 or shEXOSC2/8 cells (n=3), and four replicates for ESC and A-iPSC in each sample group (n=4). Statistical significance was determined by two-sided t-test.

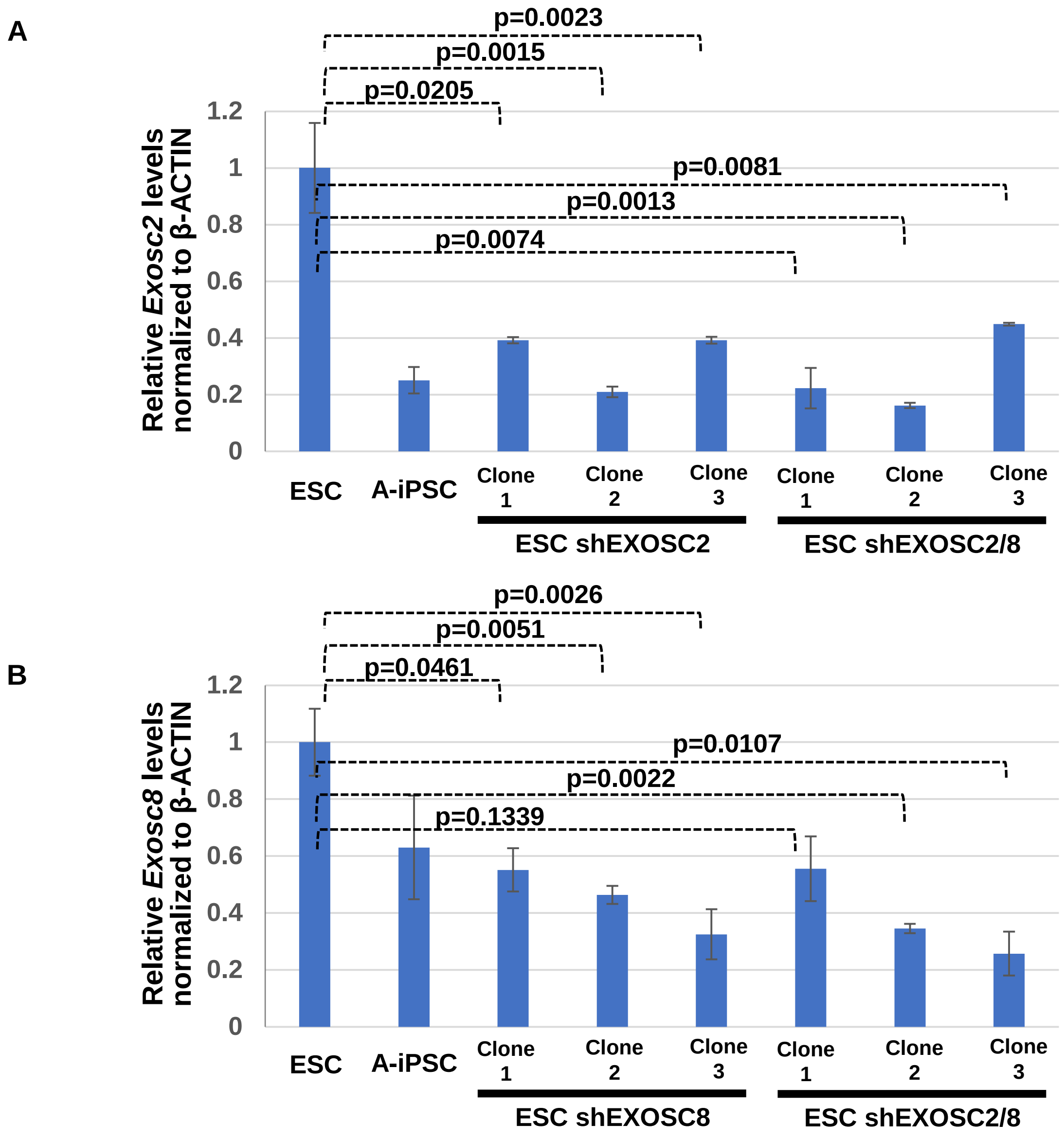

Figure S4. Clonal variability (Related to Figures 1-4). (A) Individual *Gpx2* values from microarray gene expression analysis (refer to Figure 1A). (B) Expression levels of *Exosc*1, 2, and 5. The scatter plot shows variability within each group. Colors indicate data points from the same clone. Error bars indicate standard error of the mean (refer to Figure 3B). (C) Apoptosis detected by flow cytometry in Y-iPSC with GPX2 overexpression and A-iPSC with ZSCAN10 or GPX2 shRNA expression. The scatter plot shows variability within each group. Colors indicate data points from the same clone (refer to Figure 2C). Mean ± standard deviation is plotted for multiple replicates. (D) Quantification of the reduced form of glutathione (GSH). The scatter plot shows the variability within a group. Colors indicate data points from the same clone. Mean ± standard deviation is plotted for multiple replicates (refer to Figure 4C).

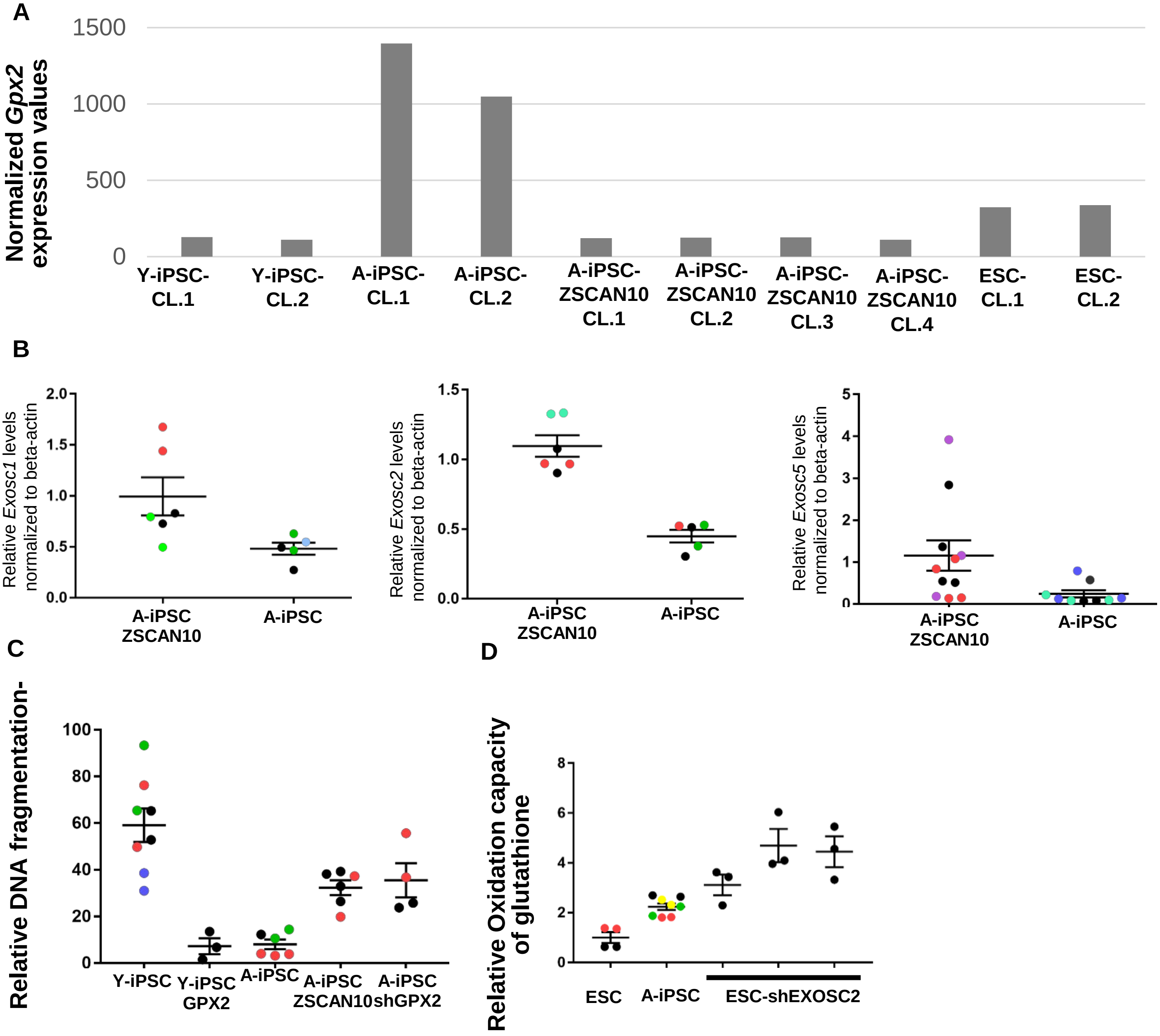

METHODS

Cell culture

Mouse ESC and iPSC were cultured in ESC media containing 20 % FBS and 1,000 U/ml of LIF (ESGRO® Leukemia Inhibitory Factor [LIF], 1 million units/1 mL). Mouse ESC were generated and their pluripotency was tested as reported in our previous publication (Kim et al., 2010). Doxycycline-inducible ZSCAN10 was induced in media supplemented with 2 µg/ml doxycycline (MP Biomedicals, doxycycline hyclate).

Generation of Y-iPSC, A-iPSC, A-iPSC-ZSCAN10

Fibroblasts were collected from B6CBAF1 mouse E17.5 embryonic skin, 5-day-old tail tip skin, and 1.5-year-old tail tip skin. Fibroblasts were not cultured for more than 3 passages to limit extensive *in vitro* culture. 10^6 fibroblasts were infected with retrovirus generated from pMX-mOCT4, pMX-mSOX2, pMX-mKLF4 (Koh et al., 2002; Moran et al., 2006) in 6-well dishes with 0.5 ml of each viral supernatant (total 2 ml per well) and spun at 2500 rpm at RT for 90 min (BenchTop Centrifuge, BeckmanCoulter, Allegra-6R). For the generation of A-iPSC-ZSCAN10 , the procedure was identical, but in addition to the four reprogramming factors, we added a doxycycline-inducible system to overexpress ZSCAN10 . This system consisted of two lentiviruses generated from a plentiRZ–ZSCAN10/plentiRZ-GPX2 and a plenti-RTTA vector (Kim et al., 2011). All cells infected with the reprogramming factors and those with additional ZSCAN10 were plated on irradiated CF-1 mouse embryonic feeder cells in a 10-cm tissue culture dish in ESC media containing 20% FBS and 1,000 U/ml of LIF. Media were changed on day 2 and doxycycline addition started on day 3 to induce ZSCAN10 overexpression. Floating cells were collected by media centrifugation and returned to culture during media changes. On day 4, cultured cells were trypsinized and replated onto four 10-cm dishes pre-coated with gelatin (0.1%) and irradiated mouse embryonic fibroblasts (MEFs) in ESC maintenance media. Media were changed daily until ESC-like colonies were observed. The reprogrammed colonies were tested for pluripotency based on teratoma formation, alkaline phosphatase staining, SSEA-1 and NANOG staining, and OCT4 expression levels.

Generation of ZSCAN10 tomato-fluorescence protein reporter by CRISPR targeting

The 5′ end of the ZSCAN10 starting codon region in ESC was targeted to insert the Tomato fluorescent protein using the CRISPR genomic targeting tool. Briefly, the left and right arms of mZSCAN10 were cloned into a plasmid PCR2.1 IRES TOMATO P2A flagbio (Invitrogen) using the following primer sets

LEFT-ZSCAN10-nhe-F:	attgctagcGAGGACTACTTGTGGAAGTCAGTG
LEFT-ZSCAN10-bamh-R:	attggatccggatccttgggagaattcaggg
RIGHT-ZSCAN10-NOT-F:	attGCGGCCGCatgctggcggaaccagtccc
RIGHT-ZSCAN10-ASC-R:	attGGCGCGCCacagacagattggacagccaggac

The resulting plasmid, along with gRNA:
CACCgATACTGCGTTAAGATCTGAC
aaacGTCAGATCTTAACGCAGTATc
CACCgTTTAGCTCCACAGGTGCAGG
aaacCCTGCACCTGTGGAGCTAAAc

The primer sets and Cas9 were transfected into ESC, and individual clones were screened for integration by PCR and DNA sequencing.

Retrovirus generation

293T cells were seeded overnight at 5×10^6 cells per 150-mm dish with DMEM supplemented with 10% FBS and penicillin/streptomycin. Retrovirus was generated using pMX-mOCT4, pMX-mSOX2, pMX-mKLF4, and pEYK-mMYC as described previously (Koh et al., 2002; Takahashi and Yamanaka, 2006). The cells were transfected with calcium phosphate as previously described (Takahashi and Yamanaka, 2006). Media were replaced with fresh DMEM twice, 18 hours after transfection. Approximately 48 hours after transfection, medium containing the retrovirus was collected and the cellular debris was removed with centrifugation. The supernatant was filtered through a 0.45-µm filter, and the retrovirus was pelleted with ultracentrifugation at 33,000 rpm in a

45Ti rotor (Beckman) for 90 min at 4°C. The retroviral particles were resuspended in ESC medium and stored at -80°C.

Lentivirus production

293T cells were seeded overnight at 5×10^6 cells per 150-mm dish with DMEM supplemented with 10% FBS and penicillin/streptomycin. The cells were transfected with plentiRZ-ZSCAN10 (mouse and human), plenti-RTTA, or plenti-GPX2, with calcium phosphate cell transfection, as previously described (Kim et al., 2011). The ZSCAN10 cDNA was clone MmCD00295052 in the pENTR223.1 backbone and the GPX2 cDNA was clone MmCD00317538 in the pCMV SPORT6 backbone from the Harvard Plasmids core (http://plasmid.med.harvard.edu/PLASMID/Home.jsp). The cDNA for mouse ZSCAN10 was subcloned into a plentiRZ vector. At 48 hours after transfection, the medium containing the lentivirus was collected and the cellular debris was removed with centrifugation. The supernatant was filtered through a 0.45-μm filter, and the lentivirus was pelleted with ultracentrifugation at 33,000 rpm in a 45Ti rotor (Beckman) for 90 min at 4°C. The lentivirus particles were resuspended in DMEM medium and stored at -80°C.

Quantitative real time-PCR (Q-PCR) analysis

The expression levels of various genes (ZSCAN10, OCT4, GPX2, EXOSC1/2/5 and β-actin) were quantified by Q-PCR. Total RNA (1 μg) was reverse transcribed in a volume of 20 μl using the M-MuLV Reverse Transcriptase system (New England Biolabs), and the resulting cDNA was diluted into a total volume of 200 μl. 10 μl of this synthesized cDNA solution was used for analysis. For pluripotency genes, each reaction was performed in a 25 μl volume using the Power SYBR Green PCR Mastermix (Applied Biosystems). The conditions were programmed as follows: initial denaturation at 95°C for 10 min followed by 40 cycles of 30 sec at 95°C, 1 min at 55°C, and 1 min at 72°C; then 1 min at 95°C, 30 s at 55°C, and 30 sec at 95°C. All of the samples were duplicated, and the PCR reaction was performed using a Mx3005P reader (Stratagene), which can detect the amount of synthesized signals during each PCR cycle. The relative amounts of the mRNAs were determined using the MxPro program (Stratagene). The amount of PCR product was normalized to a percentage of the expression level of β-actin. The PCR products of OCT4, ZSCAN10, GPX2, EXOSC1/2/5, and β-actin were also evaluated on 1.2% agarose gels after staining with ethidium bromide. The primers used to amplify the cDNA were the following:

The primers used to amplify the cDNA were the following:
OCT4-For: GGCTCTCCCATGCATTCAA and
OCT4-Rev: TTTAACCCCAAAGCTCCAGG
ZSCAN10-For: GGCTCAGAGGAATGCGTTAG and
ZSCAN10-Rev: CATCTACAGGCCCACCAGTT
GPX2-For: GTGCTGATTGAGAATGTGGC and
GPX2-Rev: AGGATGCTCGTTCTGCCCA
β-ACTIN-For: TCGTGGGTGACATCAAAGAGA and
β-ACTIN-Rev: GAACCGCTCGTTGCCAATAGT,
EXOSC2-For: CCCCAAGGAGCATCTGACAA and
EXOSC2-Rev: CCAACCCACCATTACCTCCC
EXOSC1-For: ATGGGTTGGTGATGGGCATAG and
EXOSC1-Rev: CCCATGCTGTCACTATTGGGT
EXOSC5-For: CCGATTCTACCGGGAATCACT and
EXOSC5-Rev: CTACATGGGCACAGACAGAGG

ChIP-Q-PCR

ChIP was performed according to the published protocol (Tian et al., 2012). Immunoprecipitation was performed with streptavidin beads (M280, Invitrogen). We used Q-PCR to analyze the EXOSC DNA fragments in the immunoprecipitated samples. Results are presented as fold enrichment against the negative control (log2 value), calculated by real-time Q-PCR to quantify the abundance of the DNA fragment of interest added to the ChIP reaction, with respect to the abundance of the DNA fragment found in the final immunoprecipitate. The primers used were GGTTCTTGCTTCCCATCCGA and GAGTTGGGGCTGAAAATGCG for the EXOSC1 binding region, AGCTCGCAAACCTCTCAGTG and CCCCCAAATCCTCACGTACC for the EXOSC2 binding region, GTGAGCAAAACCTGCTGTCC and AACTTGGTGAGGCTCGTACC for the EXOSC5 binding region, and GGAAACGGGTCTGAGGCTAC and CCTCGGGAGAGGTAGTGACA

for the EXOSC10 region. The ZSCAN10 binding site on the EXOSC were estimated based on published ChIP-on-Chip data (Yu et al., 2009). An Oct4 promoter region was used as a positive control (AGGAAAGAGGCCCCGGCCTT and CCAGAGAAGTGCTGGCTCTGCG) and an 80-bp genomic region on chromosome 4 was used as a negative control (GGTGGGTTACACCTCATCGG and TAACAGCACTTGTCAGGCGA).

Drug treatments

Phleomycin (Sigma) was added at 30 μg/ml for 2 hours. Cells were processed for analysis 30 min after phleomycin treatment unless indicated otherwise. After a 30-min recovery in ESC media, the cells were collected and processed for the immunoblot experiments. In the DNA fragmentation assay, the cells were given 15 hours to recover.

DNA fragmentation analysis

DNA fragmentation was measured using an *in situ* cell death assay kit (Roche) for visualization of DNA strand breaks by labelling the free 3′-OH termini with modified nucleotides (e.g., biotin-dUTP, DIG-dUTP, fluorescein-dUTP) in an enzymatic reaction. iPSC cells (1 x 10^5 cells) were treated with phleomycin (30 μg/ml) for 2 hours. Samples were collected as control or treated for analysis 15 hours after phleomycin treatment. Additionally, cells were treated with DNAase I recombinant (Roche) (10 min, 3 U/ml, at 15°C to 25°C) to induce DNA strand breaks, as a positive control for apoptosis. Medium containing floating cells and attached cells was centrifuged (1000 g, 5 min) and collected. Cells were processed for flow cytometry analysis or microscopy.

Immunoblot analysis

Treated and untreated cells (1 x 10^5 cells) were collected 30 min after the 2-hour phleomycin treatment (30 μg/ml). To harvest protein, 100-200 mL RIPA buffer (50 mM Tris- HCl [pH 7.4], 150 mM NaCl, 1% NP40, 0.25% Na-deoxycholate, 1 mM PMSF, protease inhibitor cocktail, and phosphatase inhibitor cocktail) was added to floating cell pellets and the remaining adherent cells. The samples were incubated on ice (10 min) and centrifuged (14,000 g, 10 min, 4°C). Protein concentrations were determined using a BCA protein assay kit (Pierce). Samples were adjusted to the same concentration with RIPA buffer (3000 μg/ml) and were combined with Laemmli Sample Buffer (BioRad) and β-Mercaptoethanol (Sigma) then heated at 95°C for 5 min and loaded onto a 4-15% Mini Protean TGX SDS-PAGE gel (BioRad). Samples on the SDS-PAGE gel were transferred to a 0.2-mm PVDF membrane at 100 V for 1 h, using a wet electro-transfer method (0.2 M glycine, 25 mM Tris, and 20% methanol). The membrane was blocked with 5% BSA in PBS-T (1 h at 4°C), followed by incubation with primary antibodies anti–phospho-ATM (Pierce, MA1-2020) (1:1000), anti-H2AX (Millipore, 05-636) (1:1000), anti-p53 (Leica Biosystems, P53-CM5P) (1:1000), or anti-beta actin (Cell Signaling, #4967) (1:5000) in blocking solution (5% BSA in phosphate-buffered saline containing Tween-20 [1:1000] PBS-T, overnight at 4°C). After primary antibody incubation, membranes were washed three times in PBS-T) prior to addition of secondary antibody labelled with peroxidase. Secondary antibodies were from Cell Signaling (1:10,000). ATM/H2AX negative ESC controls was imported from Xie's laboratory (Rass et al., 2013).

H_2O_2 reactive oxygen species (ROS) assay (DCFDA assay)

H_2O_2 scavenging activity was measured using a cellular reactive oxygen species assay kit (Abcam, ab113851). ESC/iPSC were labelled with 20 μM DCFDA (2',7'-dichlorofluorescein diacetate; a fluorogenic dye that measures hydroxyl, peroxyl, and other ROS activity within the cell), and cultured for 3 h with 50 μM TBHP (tert-butyl hydrogen peroxide; stable chemical form of H_2O_2). Cells were then analysed on a fluorescent plate reader. Mean ± standard deviation is plotted for four replicates from each condition.

Glutathione detection assay

Feeder-free cells were cultured on Matrigel-coated tissue culture plates in MEF-conditioned ESC-media. On day 3, the cells were washed in PBS and scraped and pelleted by centrifugation. Subsequent steps were performed using a Glutathione Fluorometric Assay Kit (cat# K264-100, Biovision Inc.) according to the manufacturer's manual. Briefly, cell pellets were homogenized in ice

cold glutathione assay buffer, preserved in perchloric acid, and centrifuged. Supernatants were neutralized with potassium hydroxide. After centrifugation, the supernatant was either used to detect reduced glutathione (GSH), or total glutathione was measured by reducing oxidized glutathione (GSSG) to GSH before measurement. For measuring GSSG concentrations specifically, existing GSH was quenched before reducing agent was applied. OPA (o-phtalaldehyde) probe, which reacts with GSH and emits fluorescence, was added to samples, and signal was acquired at Ex/Em=340 nm/420 nm on a Varioscan Flash by Thermo Scientific. Oxidation capacity of glutathione was determined by the quantity of total glutathione (GSH+GSSG).

Generation of A-iPSC-shGPX2, Y-iPSC-GPX2, ESC-shEXOSC2, ESC-shEXOSC8, and ESC-shEXOSC2&8

A-iPSC were infected post-reprogramming with a set of shRNA viruses for GPX2 (6 GIPZ Lentiviral shRNA vectors from Thermo Scientific: RMM4532-EG14776). Clones were selected with puromycin, and the levels of down-regulation were measured by Q-PCR. ESCs were infected with a set of shRNA viruses for EXOSC2 and/or EXOSC8 (2 GIPZ Lentiviral shRNA vectors for EXOSC2 from GE DHARMACON: RMM4431-200370629, RMM4431-200332733 and 3 GIPZ Lentiviral shRNA vectors for EXOSC8 from GE DHARMACON: RMM4532-EG69639). Clones were selected with puromycin. Y-iPSC were infected with a lentivirus carrying the GPX2 cDNA post-reprogramming (Harvard Plasmid Core (http://plasmid.med.harvard.edu/PLASMID/Home.jsp). The infected clones were assessed for GPX2 expression levels by Q-PCR.

GPX2 ARE reporter constructs

207 bp of a DNA fragment containing 2 AREs was amplified by PCR using mutated and wild type primer sets.

Wild type ARE primers:
attgctagcTATTATTTAAAGGCTTGTTTCTAATC
atgaattcTTAAATCATAAAGAGAAACAGAATC

Mutant ARE primers:
attgctagcTctTcTTcAAGGCTTGTTTCTAATC
atgaattcTTcAcTCATAAAGAGAAACAGAATC

In addition, NheI restriction enzyme sites were added at the 5′ end of the forward primer sets, and cloned into pGEMTeasy vector (Promega). The NheI and EcoR1 digested DNA insert was cloned into NheI and EcoRI sites of pDeEGFP-N1 (Clontech), which encodes a rapidly degraded form of GFP (half life: 2 hours). The resulting plasmids were transfected by electroporation to obtain GFP-positive ESC and control ESC-shEXOSC2. RNA stability was measured after addition of 0.2 µg/ml of actinomycin D (Casse et al., 1999) at different time points by fluorescence signal and Q-PCR of the transcript.

Electroporation of constructs

Briefly, $6x10^6$ of ESC or ESC shEXOSC2&8 were trypsinized, washed twice with PBS, and then diluted in 500 µl of EMBRYOMAX electroporation buffer (Millipore cat#ES-003-D). 40 µg of DNA of each GPX2 ARE expression construct (wild type or mutant) was added in each cell line and the mixture of cells and DNA was transferred to a Gene Pulser Cuvette (Biorad, cat# 165-2088). An exponential electroporation protocol was followed using a GENE PULSER XCELL (Biorad) with the following settings: 220 voltage (V), 950 capacitance (µF), 1000 resistance (Ω), 4 mm cuvette. The cells were then plated in a 10-mL dish with feeder MEF and allowed to recover overnight. The cells were analyzed two days later by flow cytometry. Approximately 40% of the cells were expressing the GFP, and the percentage was similar for both constructs.

Gene expression analysis

Illumina genome-wide gene expression arrays (MouseRef-8 v2.0 MouseRef-8 v2.0 BeadChip) were used for mRNA expression analysis.

Statistics and reproducibility

Data were analysed using GraphPad Prism 5.0.1 software (GraphPad Software) and/or Excel software (Microsoft Office). Data are presented as mean with s.e.m. or s.d. (as indicated in the figure legends). Statistical tests were performed and P value thresholds were obtained using

GraphPad 5.0.1. or Excel. Comparisons between two groups were performed using two-tailed unpaired Student's t-test. To confirm the reproducibility, every experiment was repeated independently at least three times.

References for Supplementary Methods and Extended Data Figures

Casse, C., Giannoni, F., Nguyen, V.T., Dubois, M.F., and Bensaude, O. (1999). The transcriptional inhibitors, actinomycin D and alpha-amanitin, activate the HIV-1 promoter and favor phosphorylation of the RNA polymerase II C-terminal domain. The Journal of biological chemistry *274*, 16097-16106.

Chu, F.F., Esworthy, R.S., Chu, P.G., Longmate, J.A., Huycke, M.M., Wilczynski, S., and Doroshow, J.H. (2004). Bacteria-induced intestinal cancer in mice with disrupted Gpx1 and Gpx2 genes. Cancer Res *64*, 962-968.

Franco, R., and Cidlowski, J.A. (2009). Apoptosis and glutathione: beyond an antioxidant. Cell death and differentiation *16*, 1303-1314.

Guo, Z., Kozlov, S., Lavin, M.F., Person, M.D., and Paull, T.T. (2010). ATM activation by oxidative stress. Science *330*, 517-521.

Harris, I.S., Treloar, A.E., Inoue, S., Sasaki, M., Gorrini, C., Lee, K.C., Yung, K.Y., Brenner, D., Knobbe-Thomsen, C.B., Cox, M.A.*, et al.* (2015). Glutathione and thioredoxin antioxidant pathways synergize to drive cancer initiation and progression. Cancer cell *27*, 211-222.

Kim, K., Doi, A., Wen, B., Ng, K., Zhao, R., Cahan, P., Kim, J., Aryee, M.J., Ji, H., Ehrlich, L.I.*, et al.* (2010). Epigenetic memory in induced pluripotent stem cells. Nature *467*, 285-290.

Kim, K., Zhao, R., Doi, A., Ng, K., Unternaehrer, J., Cahan, P., Huo, H., Loh, Y.H., Aryee, M.J., Lensch, M.W.*, et al.* (2011). Donor cell type can influence the epigenome and differentiation potential of human induced pluripotent stem cells. Nature biotechnology *29*, 1117-1119.

Koh, E.Y., Chen, T., and Daley, G.Q. (2002). Novel retroviral vectors to facilitate expression screens in mammalian cells. Nucleic Acids Res *30*, e142.

Moran, J.L., Bolton, A.D., Tran, P.V., Brown, A., Dwyer, N.D., Manning, D.K., Bjork, B.C., Li, C., Montgomery, K., Siepka, S.M.*, et al.* (2006). Utilization of a whole genome SNP panel for efficient genetic mapping in the mouse. Genome research *16*, 436-440.

Rass, E., Chandramouly, G., Zha, S., Alt, F.W., and Xie, A. (2013). Ataxia telangiectasia mutated (ATM) is dispensable for endonuclease I-SceI-induced homologous recombination in mouse embryonic stem cells. The Journal of biological chemistry *288*, 7086-7095.

Sharova, L.V., Sharov, A.A., Nedorezov, T., Piao, Y., Shaik, N., and Ko, M.S. (2009). Database for mRNA half-life of 19 977 genes obtained by DNA microarray analysis of pluripotent and differentiating mouse embryonic stem cells. DNA research : an international journal for rapid publication of reports on genes and genomes *16*, 45-58.

Skamagki M., C.C., Yeung P., Baslan T., Beck S., Zhang C., Ross C.A., Dang L., Liu Z, Giunta S., Chang T.P., Wang J., Ananthanarayanan A., Bohndorf M., Bosbach B., Adjaye J., Funabiki H., Kim J., Lowe S, Collins J.J., Lu C-W, Li H., Zhao R., Kim K. (2017). ZSCAN10 expression corrects the genomic instability of iPSC from aged donors. Nature Cell Biology *(9)*, 1037-1048.

Sleigh, M.J. (1976). The mechanism of DNA breakage by phleomycin in vitro. Nucleic acids research *3*, 891-901.

Takahashi, K., and Yamanaka, S. (2006). Induction of pluripotent stem cells from mouse embryonic and adult fibroblast cultures by defined factors. Cell *126*, 663-676.

Tian, B., Yang, J., and Brasier, A.R. (2012). Two-step cross-linking for analysis of protein-chromatin interactions. Methods in molecular biology *809*, 105-120.

Yu, H.B., Kunarso, G., Hong, F.H., and Stanton, L.W. (2009). Zfp206, Oct4, and Sox2 are integrated components of a transcriptional regulatory network in embryonic stem cells. The Journal of biological chemistry *284*, 31327-31335.
